# Pharmacogenomics of GPCR Drug Targets

**DOI:** 10.1016/j.cell.2017.11.033

**Published:** 2018-01-11

**Authors:** Alexander S. Hauser, Sreenivas Chavali, Ikuo Masuho, Leonie J. Jahn, Kirill A. Martemyanov, David E. Gloriam, M. Madan Babu

**Affiliations:** 1 MRC Laboratory of Molecular Biology, Francis Crick Avenue, Cambridge CB2 0QH, UK; 2 Department of Drug Design and Pharmacology, University of Copenhagen, Universitetsparken 2, 2100 Copenhagen, Denmark; 3 Department of Neuroscience, The Scripps Research Institute Florida, Jupiter, FL 33458, USA; 4 The Novo Nordisk Foundation Center for Biosustainability, Technical University Denmark, Kemitorvet 2800 Kgs. Lyngby, Denmark

**Keywords:** GPCR, pharmacogenomics, FDA approved drugs, clinical trial, drug response, natural variation, polymorphism, economic burden, opioid receptor, personalized medicine

## Abstract

Natural genetic variation in the human genome is a cause of individual differences in responses to medications and is an underappreciated burden on public health. Although 108 G-protein-coupled receptors (GPCRs) are the targets of 475 (∼34%) Food and Drug Administration (FDA)-approved drugs and account for a global sales volume of over 180 billion US dollars annually, the prevalence of genetic variation among GPCRs targeted by drugs is unknown. By analyzing data from 68,496 individuals, we find that GPCRs targeted by drugs show genetic variation within functional regions such as drug- and effector-binding sites in the human population. We experimentally show that certain variants of μ-opioid and Cholecystokinin-A receptors could lead to altered or adverse drug response. By analyzing UK National Health Service drug prescription and sales data, we suggest that characterizing GPCR variants could increase prescription precision, improving patients’ quality of life, and relieve the economic and societal burden due to variable drug responsiveness.

**Video Abstract:**

## Highlights

• GPCRs targeted by FDA-approved drugs show genetic variation in the human population
• Genetic variation occurs in functional sites and may result in altered drug response
• We present an online resource of GPCR genetic variants for pharmacogenomics research
• Understanding variation in drug targets may help alleviate economic healthcare burden

## Graphical Abstract

**Figure:**
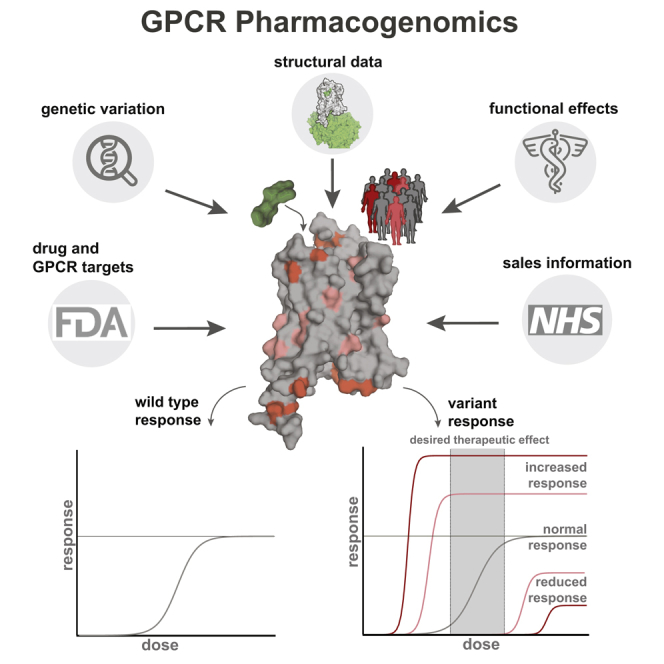

## Teaser

A pharmacogenomic analysis probes the effects of genetic variation in G-protein-coupled receptors on responses to FDA-approved drugs and estimates how this genetic variation may impact healthcare costs.

## Introduction

A system of rigorous clinical trials and regulation exist to ensure that a new drug is safe and effective when reaching the market. However, natural human genetic variation(s) may cause individuals to respond differently to the same medication. For instance, genetic variation is linked to differences in response to anti-hypertensive drugs such as β-blockers, angiotensin receptor blockers, and angiotensin converting enzyme (ACE) inhibitors (Johnson, 2008, Liggett et al., 2006, Mialet Perez et al., 2003). Natural variation may also increase the propensity for adverse reaction to drugs (Roden and George, 2002). Thus, genetic variation in drug targets may alter therapeutic efficacy and safety of drugs. Inadequate accounting for adverse drug reactions cost a fiscal burden of ∼30 billion US dollars annually in the US alone (Sultana et al., 2013). Hence, understanding genetic variation in drug targets has direct bearing on tailoring drug prescriptions (i.e., personalized healthcare) to maximize efficacy and safety while reducing side effects.

**Figure 1:**
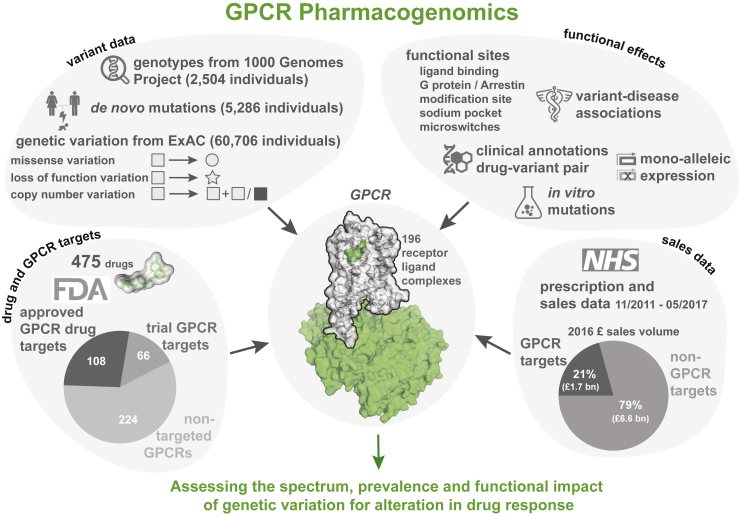
Pharmacogenomic Landscape of GPCR Drug Targets Schematic highlighting the different types of data analyzed in this study, ranging from data on drug targets, variants, functional effects, sequences, structures to information on prescription, and sales of drugs in the UK.

Many druggable targets for treatment of common diseases involve G-protein-coupled receptors (GPCRs) that mediate therapeutic effects of ∼34% of the marketed drugs (Santos et al., 2017, Rask-Andersen et al., 2014, Hauser et al., 2017). The sales of GPCR targeting drugs represent a global market share of over 27% (The IDG Knowledge Management Center, 2016). Food and Drug Administration (FDA)-approved drugs target at least 108 GPCRs (herein referred as GPCR drug targets), with an additional 66 receptors targeted by agents that are/were in clinical trials (Table S1). Some drugs act through several targets and frequently include GPCRs. Thus, GPCRs serve as primary and secondary targets and determine the pharmacological profiles of the responses (Allen and Roth, 2011, Hauser et al., 2017). Although studies have identified polymorphisms in GPCRs that lead to variable or adverse drug response (Table S2), the prevalence and impact of genetic variation among all human GPCRs that are targeted by FDA-approved drugs remain unknown. In this study, we present a comprehensive analysis and map of the pharmacogenomics landscape of GPCR drug targets (Figure 1).

### Prevalence and Incidence of Natural Variation in GPCR Drug Targets

**Figure 2:**
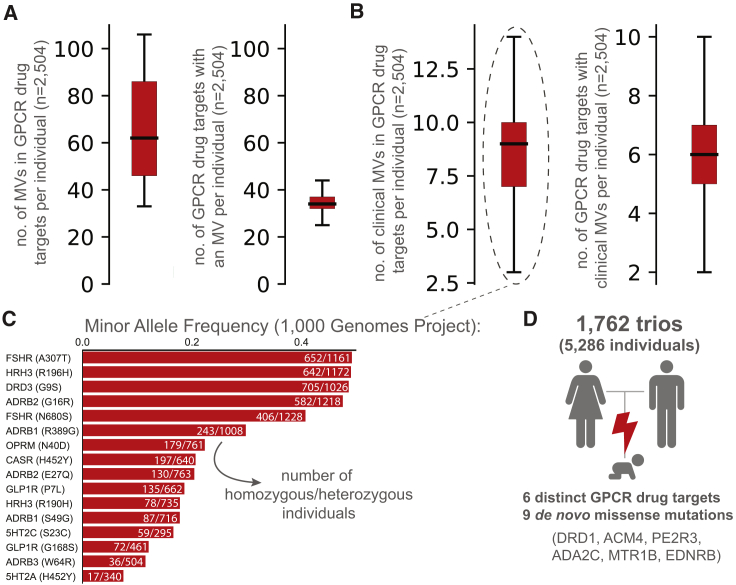
Distribution of Individuals Harboring Missense Variation in GPCR Drug Targets (A and B) Estimates based on genotype data from 2,504 individual genomes was made per individual on (A) number of missense variants in GPCR drug targets (left) and the number of GPCR drug targets harboring a missense variation (right) and (B) number of clinically known variants that alter efficacy of drug response or toxicity in GPCR drug targets (left) and the number of affected GPCR drug targets with clinically known mutations (right). (C) Allele frequencies of variants, known to alter drug response in 2,504 individuals (number of homozygous/heterozygous carriers) (Table S2). (D) Analysis of 1,762 studied trios (father-mother-offspring) revealed a total of 9 *de novo* missense mutations in 6 GPCR drug targets.

What is the prevalence of GPCR drug targets to harbor a missense variation (MV) within an individual? An investigation of complete genotype information for 2,504 “healthy” individuals from the 1000 Genomes Project (Auton et al., 2015) showed that, on average, an individual harbors 68 missense variations within the coding region of one-third of the GPCR drug targets (Figure 2A). Of these, an average of 8 variants per individual have previously known clinical associations with altered drug response (Figure 2B). For instance, the heterozygous A307T variation (minor allele frequency [MAF]: 0.49) in the follicle stimulating hormone receptor (FSHR) is prevalent in women who develop polycystic ovary syndrome and is associated with a higher responsiveness to exogenous FSH (Dolfin et al., 2011). The G9S variant (MAF: 0.48) in the dopamine receptor 3 (DRD3) is linked to increased risk of gastrointestinal toxicity upon Levodopa treatment in subjects with Parkinson’s disease (Rieck et al., 2016) (Figure 2C). Analysis of 1,762 trios (father, mother, offspring) (Turner et al., 2017) revealed that 6 offsprings harbor at least one new *de novo* MV in a GPCR drug target. In other words, a new, non-lethal, missense, germline mutation in a GPCR drug target arises in 1 of every ∼300 newborns (Figure 2D). These observations collectively suggest that GPCR drug targets are likely to show substantial variation with new missense mutations continuing to arise within their coding region.

### Mutational Landscape of GPCR Drug Targets

In addition to MV, mutations that introduce a stop codon, cause a frameshift or affect essential splice sites constitute loss-of-function variations (LoF). The abundance of a protein-coding gene may be affected by deletions and/or duplications (copy number variation [CNV]). Such mutational events may alter the functional property and/or change the abundance of a drug target, either of which can influence drug efficacy, safety profile, and adverse reaction. How much variability is seen in the GPCR drug targets in the human population? To characterize the spectrum and prevalence of variation in GPCR drug targets, we investigated data from the exome aggregation consortium (ExAC), which contains aggregated information on MVs, LoFs, and CNVs for ∼60,000 ‘healthy’ individuals (Lek et al., 2016, Ruderfer et al., 2016). This allowed us to characterize the mutational landscape of currently druggable GPCRs in the human population.

**Figure 3:**
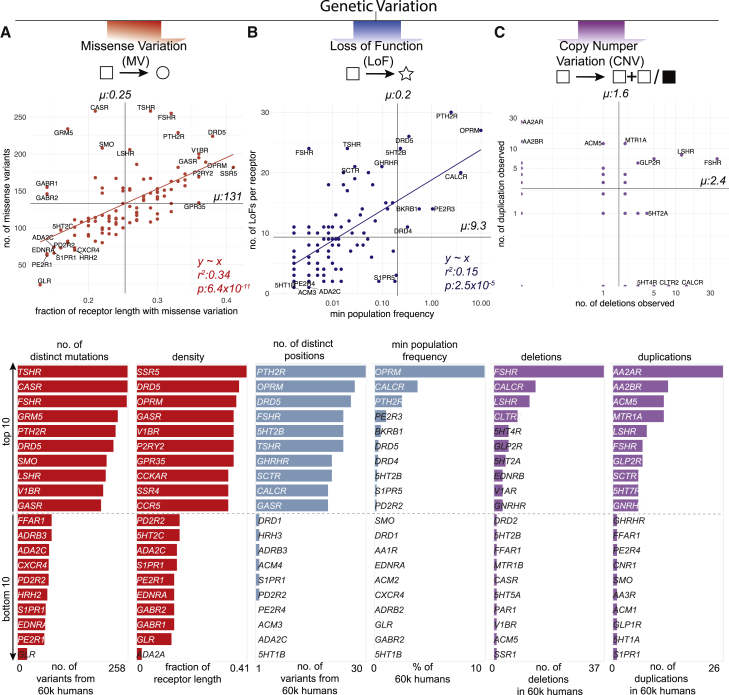
Genetic Variation Landscape of GPCR Drug Targets (A–C) Scatterplots of (A) missense variation (red), (B) loss-of-function mutations (blue), and (C) copy-number variation (purple) for GPCR drug targets. Each mutation type shows the number of observed variants (separated into deletions and duplications for CNVs) for a given GPCR drug target. Missense variation density was obtained by normalizing number of missense mutations to the receptor sequence length. Loss-of-function mutations are presented as the minimum percentage of individuals harboring at least one copy of a protein-truncating variant (STAR Methods). Correlations and mean values (μ) are shown for MVs and LoFs. Mean values (μ) for the distributions are provided. Genetic variation landscapes of GPCR drug targets that are in clinical trials are provided in Table S3 and S4). Lower half of the figure shows the distribution of top 10 (upper panels) and bottom 10 ranking GPCR drug targets (lower panels). See also Figures S1, S2, and S3.

**Figure S1:**
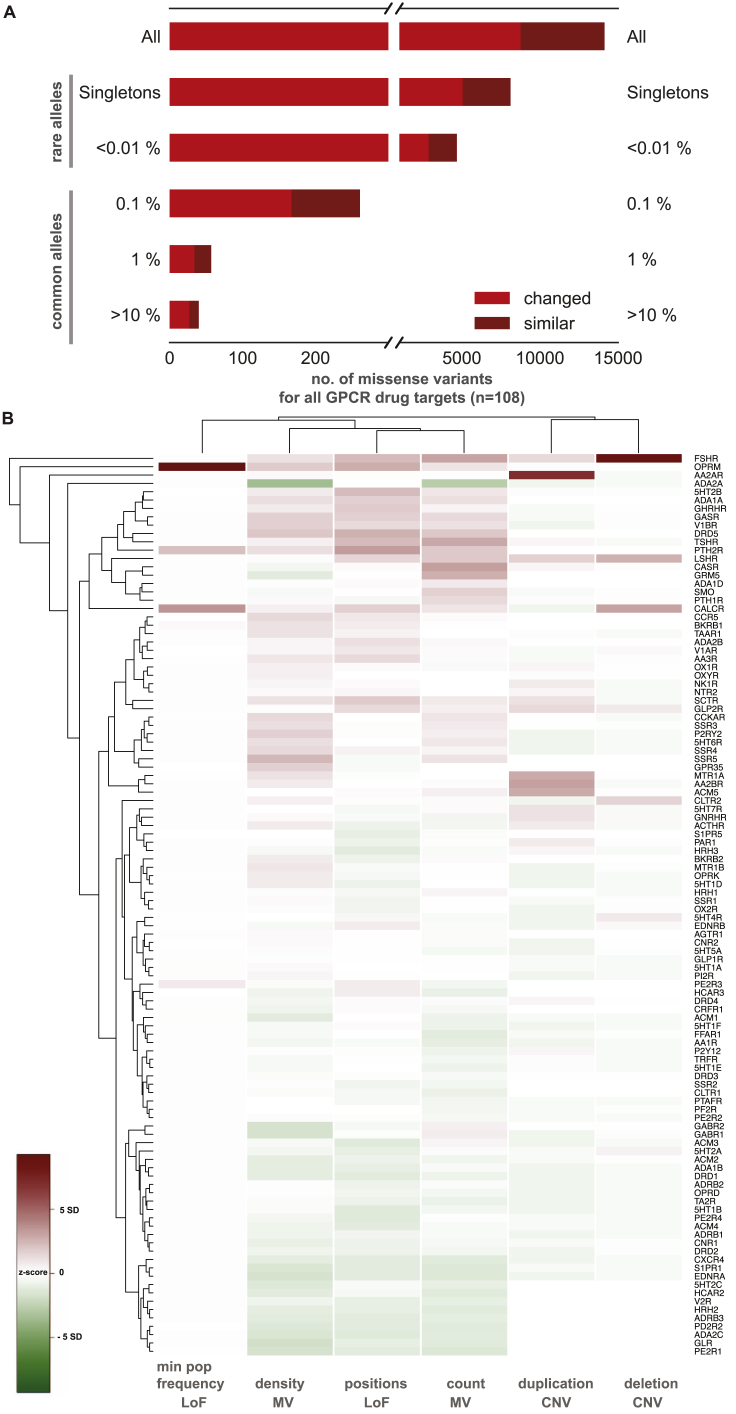
Frequency of Genetic Variants in GPCR Drug Targets, Related to Figure 3 (A) The allele frequency spectrum (ExAC data) of the 108 GPCR targets of approved drugs shows that most genetic variants are rare (single observations or allele frequency ≤ 0.01%). Common variants (> 0.01%) exist for 358 sites. The coloring shows missense variations with an amino acid property change (‘changed’) and missense variations, where the mutant amino acid substitution is within the same class of amino acid properties (‘similar’). (B) To assess the most polymorphic GPCR drug targets (*rows*) across different categories including for MVs, LoFs and CNVs, Z-scores were calculated within categories (*columns*) and grouped by hierarchical clustering. Receptors with high genetic variation are highlighted in red (Table S3).

We find a total of 14,192 MVs in 108 GPCR drug targets, with a mean of 128 rare (MAF <1 × 10^−3^) and 3.7 common (MAF ≥ 1 × 10^−3^) variants per receptor (Figure 3A and S1A). On average, 25% of all positions in each of the 108 GPCRs contain a MV (Figure 3A). GPCR drug targets have, on average, a LoF mutation in 9.3 different positions per receptor (Figure 3B). Our conservative estimate suggests that on average, at least 120 of the 60,706 individuals harbor such LoF mutations (i.e., stop codon, essential splice site, and frameshift mutation) in a GPCR drug target (0.2%; STAR Methods). In fact, a minimum of one LoF variant has been observed in each of the 108 GPCRs suggesting that heterozygosity, regulatory epistasis, and buffering mechanisms such as allele-specific expression might offset the effects of these drastic mutations in healthy individuals (Lappalainen et al., 2011, Kukurba et al., 2014). Many GPCR drug targets are also susceptible to CNVs and each of the GPCRs analyzed had on average two duplications and three deletions reported in the ExAC dataset (Figure 3C).

**Figure S2:**
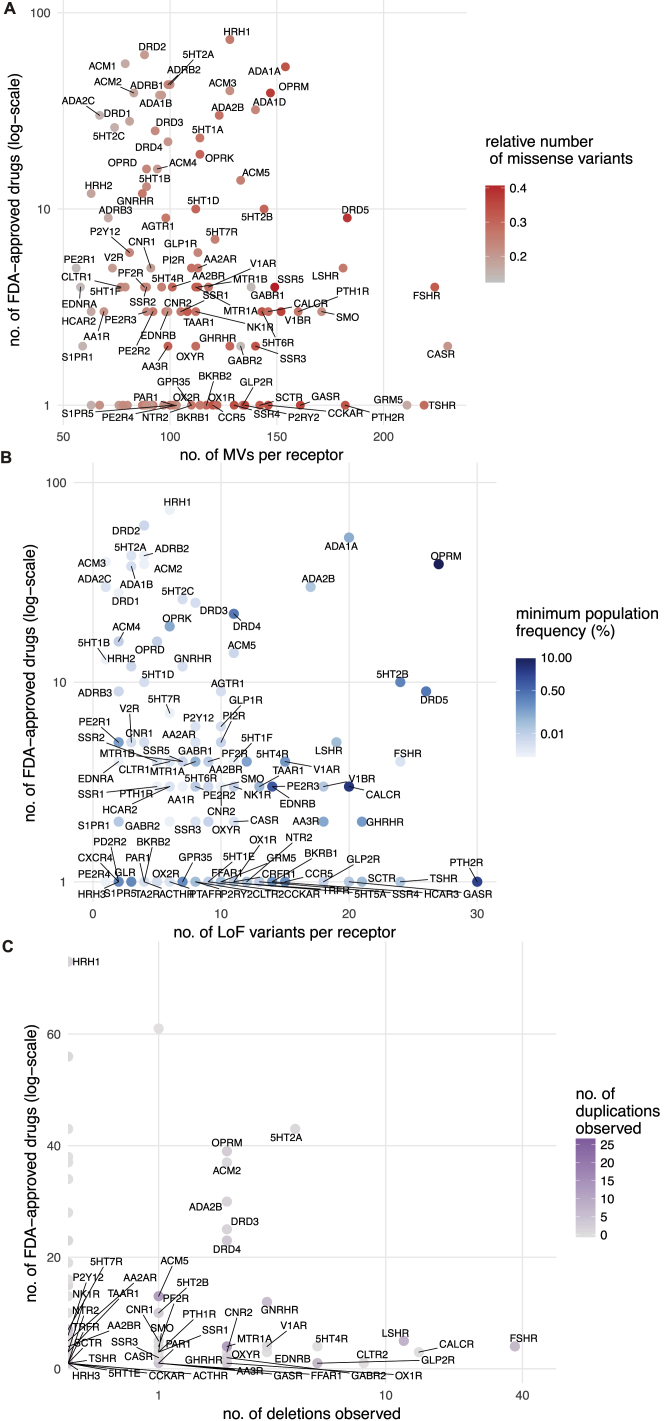
Loss-of-Function and Copy-Number Variations in GPCR Drug Target, Related to Figure 3 (A–C) Distribution of human GPCRs by the number of FDA approved drugs that target them (*y axis, logarithmic scale*) and (A) the number of missense variants (*x axis*) along with the fraction of MVs by receptor length (red color scale), (B) the number of loss of function variants (*x axis*) along with a conservative estimate of the minimum population frequency (blue color scale) and (C) the number of observed deletions (*x axis*) and duplications (purple color scale). GPCRs that are frequently targeted by drugs (i.e., many FDA-approved drugs) are highly polymorphic in terms of MVs, LoF variants and CNVs.

The μ-opioid receptor (MOR; OPRM1), targeted by analgesics, is one of the highly variable GPCR drug targets in the human population (Table S3; Figure S1B). Integrating the information about the extent of variability of GPCR targets with the FDA-approved drugs revealed that several of the highly polymorphic GPCRs are targeted by a large number of drugs (Figures S2A–S2C). Thus, the extensive genetic variation in GPCR drug targets may contribute to a substantial, and as yet underappreciated, variability in drug responses between individuals in the population.

### Putative Functional Impact of Variants in GPCR Drug Targets

**Figure 4:**
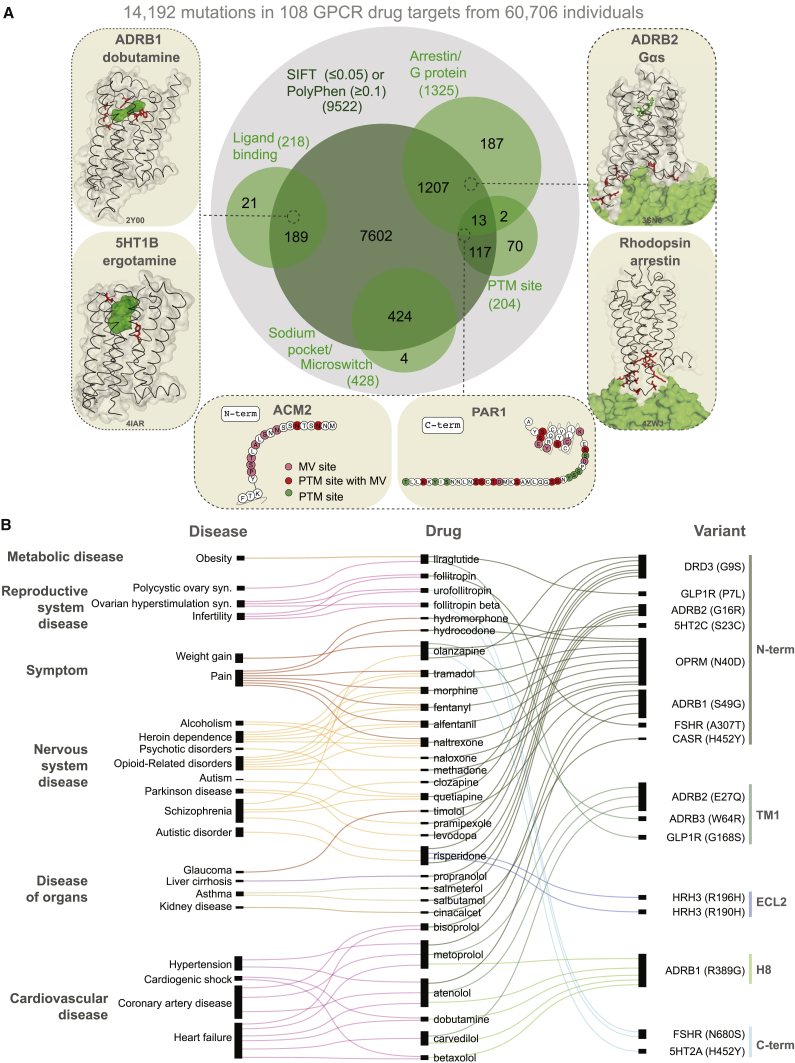
Missense Variations in GPCR Drug Targets and Their Possible Functional Impact (A) Variants predicted to have impact by SIFT or PolyPhen (dark green). MVs can affect different functional sites (light green), which were defined as ligand binding (left), post-translational-modification site (bottom), and micro-switches including allosteric sodium ion binding pocket and G-protein/arrestin interaction interface (right). The displayed structures show missense variants within 5 Å (red) of an approved drug (left and right) or MVs within 5 Å distance to the G protein or arrestin. PDB IDs are provided in the bottom of each structure sub-panel. (B) Disease ontology (left), FDA-approved drug (middle), and variant (right) known (i.e., statistical association in clinical-genetics studies) to alter drug response or efficacy or lead to adverse drug response (Table S2). In some cases, the drug and disease are linked to reflect the clinical study design and are not drugs given to treat those diseases. See also Figure S4.

Can the observed variants of a receptor affect drug response and what fraction of the variants is likely to have an impact? We analyzed each of the MVs and investigated whether they map to functionally relevant regions (i.e., ligand binding, effector binding, allosteric sodium binding pocket, activation micro-switches, and post-translational modification sites) to infer possible impact (Figure 4A; Table S4). We identified functional sites based on data from all 196 available GPCR-ligand crystal structures; published literature and 2,544 experimentally validated post-translational modification sites (PTMs). 2,036 mutations in the different receptors fall within known functional sites (14.3% of 14,192 MVs; *Z* score = 3.7; p = 1.5 × 10^−4^; permutation test; Figure 4A; Table S4).

**Figure S3:**
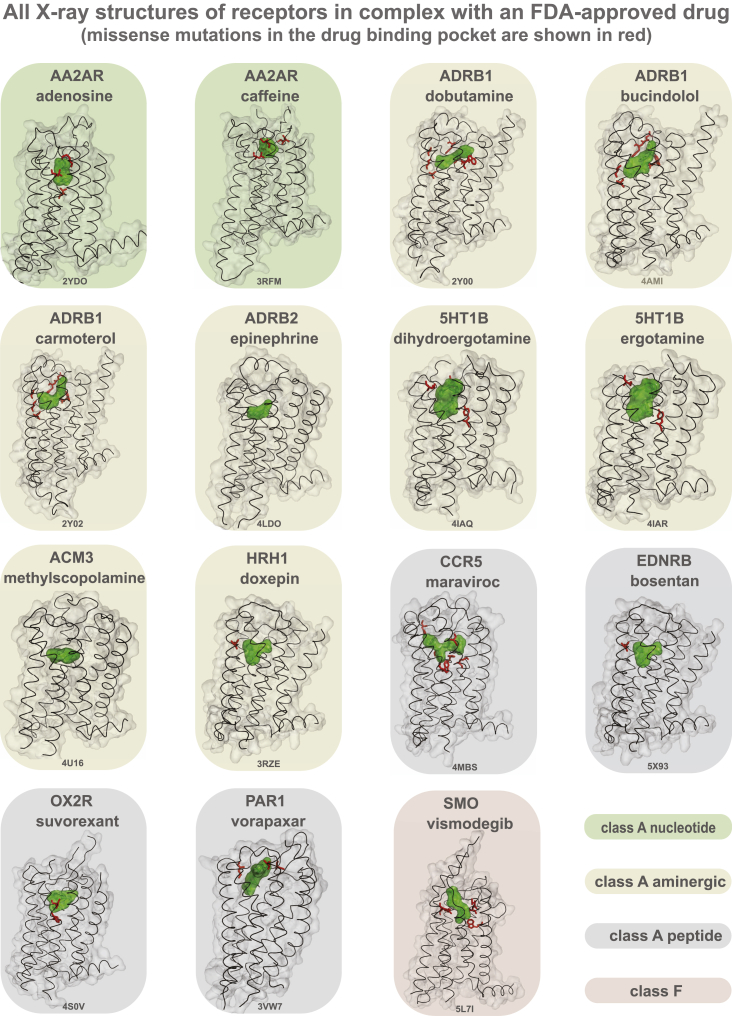
All Available X-Ray Crystal Structures of GPCRs in Complex with FDA-Approved Drugs, Related to Figure 4 FDA approved drugs bound to their respective receptors (n = 15) are shown in green. Missense variations from 60,706 individuals within 5Å of the co-crystallized drug are highlighted in red.

Examination of structures of GPCRs bound to FDA-approved drugs revealed missense variation in the drug-binding pocket of several receptors (Figure S3). For instance, 8 of the 9 positions in the drug-binding pocket of maraviroc (antiretroviral drug) in the CCR5 receptor exhibit polymorphism suggesting that patients carrying such variant CCR5 receptors may show altered response when treated for HIV infection. One in every 10 MV resides in the G-protein- or β-arrestin-binding interface (Table S4). For instance, of the 108 GPCR drug targets, we find that for 67 receptors, there is at least one allele with a mutation in the highly conserved 3.50×50 position (GPCRdb numbering) and for 41 receptors in the position 8.49×49. These positions make extensive non-covalent contacts with G-protein and arrestin, respectively (Kang et al., 2015, Liang et al., 2017, Rasmussen et al., 2011, Zhang et al., 2017) and hence are important for intracellular signaling and drug response. This suggests that individuals with such receptor variants may exhibit differences in G-protein selectivity or biased signaling and thus respond differently to the same drug due to differences in GPCR signaling.

**Figure S4:**
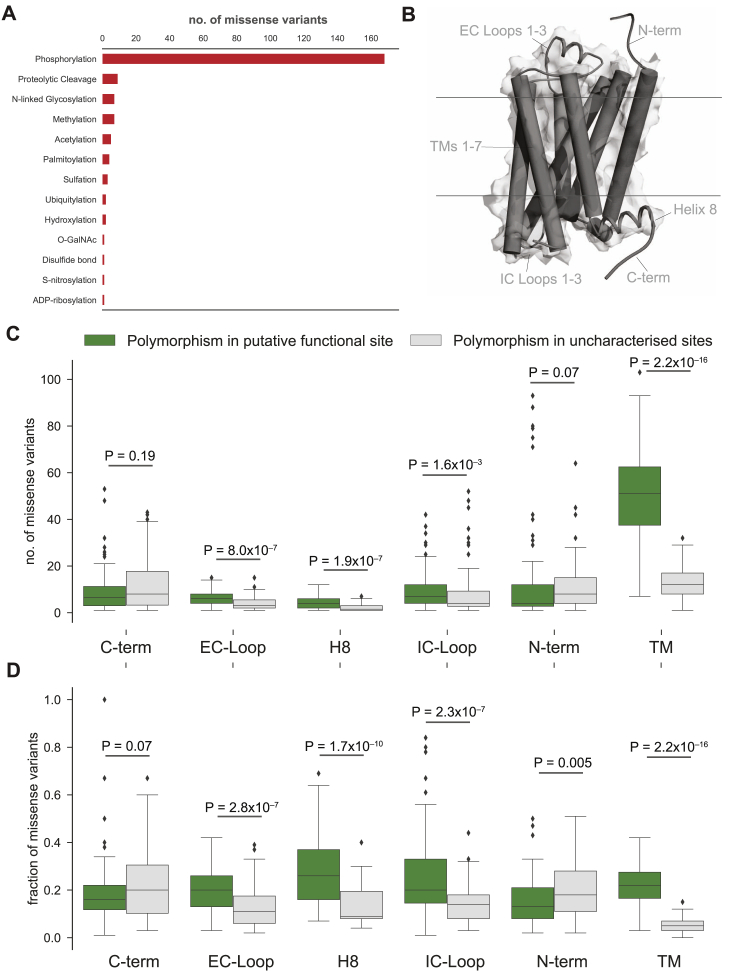
Missense Variants in Post-translational Modification Sites and Structural Segments, Related to Figure 4 (A) Missense variants were mapped onto experimentally verified post-translational modification sites of GPCR drug targets (n = 846). Number of missense variants per post-translational modification site type. (B) Structural segments were assigned for each receptor. Each segment was then aggregated into higher-order groups: C terminus, extracellular loops, transmembrane region, intracellular loops, helix 8 and N terminus (*top*). Cartoon representation of the β_2_AR (PDB: 2RH1). (C) Missense variants were projected onto each structural segment. Variants that are predicted to have a functional impact map (green) significantly more often into the transmembrane region and loops (Wilcoxon rank sum test; EC-Loops: p < 8.0x10^−7^, IC-Loops: p < 1.6x10^−3^, TM: p < 2.2x10^−16^) variants of unknown functional impact (gray). (D) Missense variants were projected onto each structural segment and normalized by segment length. (Wilcoxon rank sum test; EC-Loops: p < 2.8x10^−07^, IC-Loops: p < 2.3x10^−7^, TM: p < 2.2x10^−16^).

Missense variants were also observed in other functional sites that influence GPCR structure, dynamics, activation, allostery, and function. These include mutations in the allosteric sodium-binding pocket that modulate receptor activity (Katritch et al., 2014), micro-switches that are critical for receptor conformational changes (Trzaskowski et al., 2012, Venkatakrishnan et al., 2016, Wacker et al., 2013), as well as post-translational modification sites such as N-glycosylation in the N-terminal tail and phosphorylation in the C-terminal tail that can influence receptor expression and signaling, respectively (Venkatakrishnan et al., 2014) (Table S4; Figure S4A). Individuals carrying such variant receptors may respond differently to drugs because of altered basal activity and signaling due to perturbation of residues important for conformational changes. They may also show differences because of altered expression, localization, trafficking, or desensitization due to disruption of residues important for key post-translational modifications (e.g., phosphorylation sites).

Analysis of the putative functional impact of these variants by SIFT and PolyPhen revealed that 9,522 of the 14,192 MVs have the potential to cause a functional impact due to changes in the physicochemical properties of the variant (Figure 4B; herein referred to putative functional sites). Of these, 1,772 MVs map to the 2,036 known functional sites described above (i.e., ∼87% of the known functional site variations have been captured by SIFT or PolyPhen; permutation test; *Z* score = 24.5; p < 1 × 10^−5^). MVs with putative functional impact map more often within the transmembrane region and loops compared to variants of unknown functional impact (Wilcoxon rank-sum test for normalized segment lengths; TM segments: p < 2.2 × 10^−16^; extracellular (EC) loops: p < 2.8 × 10^−7^; intracellular (IC) loops: p < 2.3 × 10^−3^; Figures S4B–S4D). It is likely that some of these variations may influence receptor conformation, dynamics, and signaling by affecting allosteric sites, effector selectivity sites, receptor stability, and oligomerization (Congreve et al., 2017, Costa-Neto et al., 2016, Flock et al., 2015, Flock et al., 2017, Gahbauer and Böckmann, 2016, Katritch et al., 2014, Venkatakrishnan et al., 2013, Venkatakrishnan et al., 2016, Wacker et al., 2017).

### Pharmacological and Clinical Effects of Receptor Variants

Are the individuals with variant receptors likely to respond differently to drugs? To investigate this, we compiled experimental data on ligand-mutant GPCR interactions. Consistent with the findings above, of the 49 experimentally tested mutations corresponding to naturally occurring variants in 24 receptors, 32% (16 of 49) show at least a 5-fold change in affinity or potency to one of the ligands tested. Of these, 68% (11 of 16) fall within known or putative functional sites (Table S4). These observations suggest that naturally occurring variations in these drug targets can affect drug binding.

We then analyzed data on drug-missense variant associations from population-based clinical studies and compiled a dataset of statistical association between mutations in 16 different positions among 11 receptors and altered response to one of 39 approved drugs (Figure 4B; Table S2). These drugs cover treatment of diverse disorders ranging from metabolic, respiratory, nervous, and reproductive system disorders to cardiovascular diseases. We categorized the diseases for which a drug is administered and linked this information with the variant, and the position on the receptor. Of the 16 variants that have been associated with an altered drug response, four variants reside within putative functional sites and have modest to high allele frequencies (MAF = 0.18 – 0.49; Figure 4B). The rest of the MVs reside outside of functional sites, suggesting that such mutations may be affecting yet-to-be characterized functional sites. Such sites may facilitate interactions with other factors such as chaperones, membrane proteins, cytosolic proteins, and membrane lipids, form a part of oligomerization interface or linear motifs within disordered regions, or may influence receptor biogenesis and expression homeostasis (Chen et al., 2014, van der Lee et al., 2014, Venkatakrishnan et al., 2014, Boucrot et al., 2015, Gupta et al., 2017).

### Natural Receptor Variants Can Impact Drug Response and Bias Signaling

**Figure 5:**
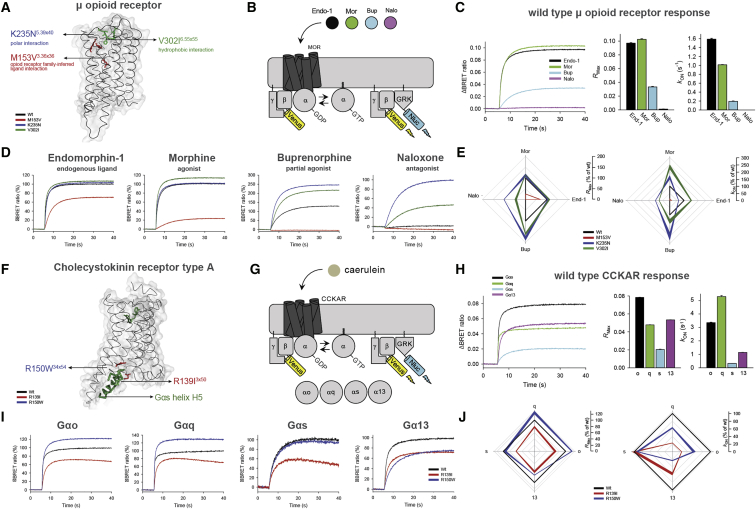
Effects of Natural Mutations on Drug Activity and G-Protein Coupling (A) Positions of selected missense variations of the μ-opioid receptor (MOR) near the ligand-binding pocket. (B) Schema of the BRET assay for real-time monitoring of G-protein activation. Activating μ-opioid receptors by agonist leads to the dissociation of inactive heterotrimeric G proteins into active GTP-bound Gα and Venus-Gβγ subunits. The free Venus-Gβγ then interacts with the Gβγ effector mimetic masGRK3ct-Nluc to increase the BRET signal. (C) Ligand/drug-induced maximum BRET amplitude (*R*_Max_) and activation rate constants (*k*_ON_) by wild-type μ-opioid receptor (mean ± SEM, n = 6 wells). (D) Real-time monitoring of ligand/drug actions on μ-opioid receptor mutants (mean response trace, n = 3 or 6). (E) Quantification of stimulus bias (*R*_MAX_, left and *k*_ON_, right) of μ-opioid receptor mutants. The values of agonist-induced responses were normalized to the reference, wild-type μ-opioid receptor (black line). The values of naloxone-induced responses were normalized to the K235N^3.36x36^ mutant (thickness represents the SEM over n = 3). (F) Positions of selected missense variations of the Cholecystokinin receptor type A. (G) Schema of the BRET assay for real-time monitoring of G-protein activity for CCKAR experiments. (H) Agonist-induced maximum BRET amplitude (*R*_Max_) and activation rate constants (*k*_ON_) by wild-type CCKAR (mean ± SEM, n = 6 wells). (I) Real-time monitoring of G-protein activation by CCKAR mutants stimulated with caerulein (30 μM, applied at 5 s, n = 3) normalized to the maximum amplitude of the wild-type receptor. (J) Quantification of G-protein-coupling bias (*R*_MAX_, left and *k*_ON_, right) of CCKAR mutants normalized to wild-type CCKAR (black line, thickness represents the SEM over n = 3). See also Figure S5.

**Figure S5:**
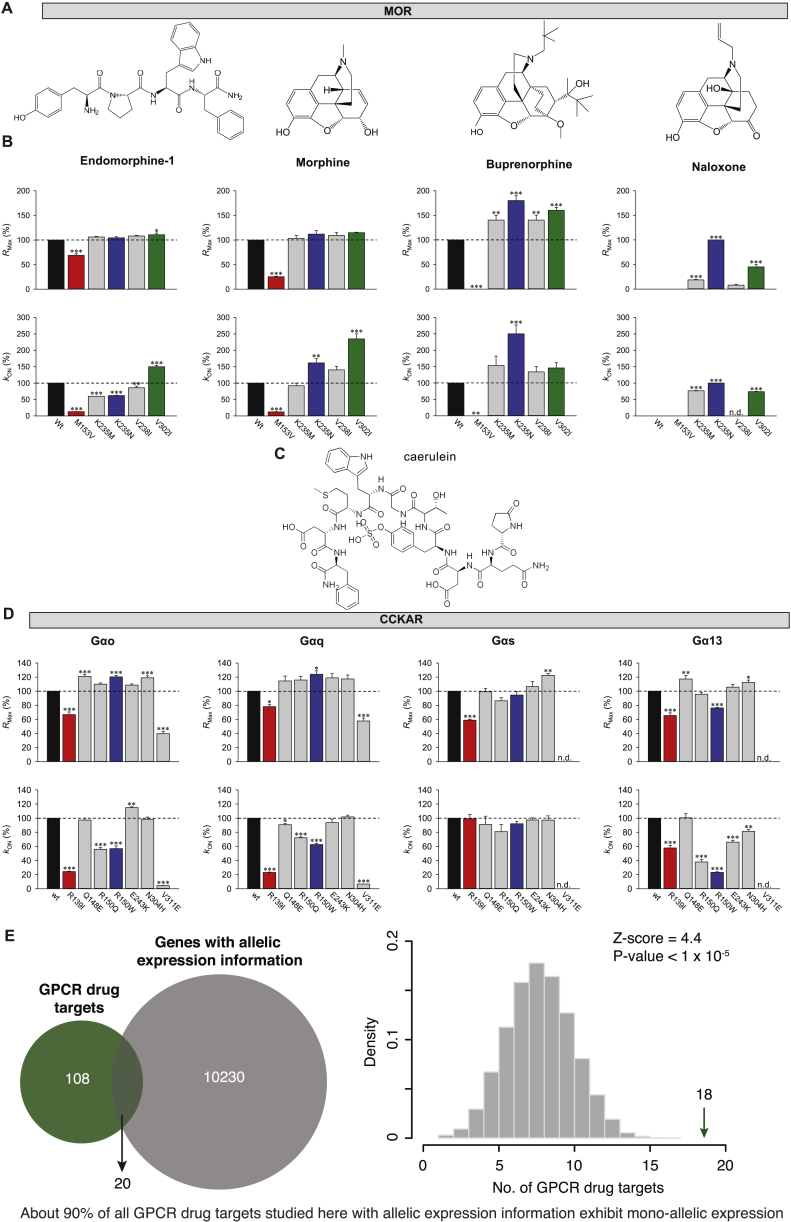
Effects of Mutations on *R*_Max_ and *k*_ON_ for μ-Opioid Receptor and CCKAR and Allele-Specific Expression of GPCR Drug Targets, Related to Figure 5 (A) Chemical structures of opioid receptor ligands. (B) The bar graphs quantitate the relative *R*_Max_ and *k*_ON_ of μ-opioid receptor mutants to μ-opioid receptor wild-type. (C) Chemical structure of Cholecystokinin receptor ligand caerulein. (D) The bar graphs quantitate the relative *R*_Max_ and *k*_ON_ of CCKAR mutants to CCKAR wild-type. For both panels, results are expressed as the mean ± SEM. One-way ANOVA with Dunnett post hoc multiple comparison test relative to “wild type receptor,” ^∗^p < 0.05, ^∗∗^p < 0.01, ^∗∗∗^p < 0.001, n = 3 independent experiments. (E) Enrichment of GPCR drug targets among genes with mono-allelic expression. The Venn diagram shows the overlap between GPCR drug targets and the genes with allelic expression data. Enrichment was tested with permutation tests by performing 100,000 iterations. The random expectation (gray histogram) and the actual observation (green arrow) of GPCRs with mono-allelic expression are shown on the right.

In order to better understand the implications of the observed variations, we experimentally investigated the impact of variations in two GPCR drug targets. We first analyzed the μ-opioid receptor, a highly variable GPCR drug target with one of the highest numbers of FDA-approved drugs (Figure S2). Previously uncharacterized polymorphisms around the ligand-binding site were selected and tested using a real-time bioluminescence resonance energy transfer (BRET) assay, which monitors state transitions in G-protein subunits upon receptor stimulation. Quantifying the onset kinetics of G-protein activation by GPCRs (K_ON_) in this assay approximates potency, while the maximal response amplitude (R_Max_) reflects the efficacy (Masuho et al., 2015a) (Figures 5A, 5B, S5A, and S5B). Our assay could reliably discriminate differential effects of full agonist (morphine) from that of partial agonist (buprenorphine) and antagonist (naloxone), compared to the endogenous agonist endomorphine-1 for the wild-type receptor (Figure 5C).

Three of the variants exhibited a range of functional alterations in μ-opioid receptor signaling (Figures 5D and 5E). One variant (M153V^3x36^) resulted in partial loss of function, reducing its responses to both full agonists and the partial agonist. The two other variants uniquely altered μ-opioid receptor pharmacology by biasing drug action in a drug selective fashion. Specifically, the K235N^3x40^ variant behaved like the wild-type receptor in response to the endogenous ligand. However, K235N^3x40^ displayed increased efficacy and potency to buprenorphine relative to the wild-type. K_ON_ estimates show that the potency of responses to full agonist morphine also increased, but that of endomorphine-1 decreased with no change in their efficacies (R_Max_). Such behavior is expected to produce little difference in baseline phenotypes in individuals carrying this mutation but would be expected to augment responsiveness to treatment with synthetic opioids, increasing the risk of an inadvertent overdose. Another variant V302I^6x55^ exhibited gain-of-function effects with increased potencies to full agonists and efficacy of the partial agonist. Importantly, both gain-of-function variants maintained G-protein signaling when treated with an antagonist naloxone, an activity not seen with wild-type human μ-opioid receptor but previously reported for the mouse receptor (Masuho et al., 2015a). These findings suggest that individuals with this variant receptor may manifest loss of efficacy when treated with naloxone, which is typically administered as a detoxifying agent in patients with opioid overdose. Further adverse reactions might arise in response to combinatorial medications, e.g., buprenorphine/naloxone if prescribed for management of opioid abuse (Boyer, 2012, Orman and Keating, 2009). Thus, receptor variants can have specific effects on the response to some drugs that may not be evident from their response to natural ligand.

We also examined changes in G-protein-binding specificity by investigating the effect of polymorphisms at the G-protein-binding interface in the cholecystokinin A receptor (CCKAR). This receptor couples to several G proteins to manifest its physiological effects (Dufresne et al., 2006) (Figures 5F, 5G, S5C, and S5D). Wild-type CCKAR robustly coupled to all four Gα subfamilies (R_Max_ in Figure 5H; for order of preference, see k_ON_ in Figure 5H). Notably, two polymorphisms in the G-protein-coupling interface (R139I^3x50^ and R150W^34x54^) differentially altered coupling preference (Figures 5I, 5J, S5C, and S5D). The R150W^34x54^ variant showed increased signaling efficacy via Gαo and Gαq, but diminished signaling efficacy via Gα13 with no alteration in Gαs coupling. In contrast, this variant exhibited diminished potency of Gαo, Gαq, and Gα13 but not Gαs activation. On the other hand, the R139I^3x50^ variant showed equally diminished efficacy across all four G proteins, yet potency was diminished only for Gαo, Gαq, and Gα13 but not for Gαs. Thus, polymorphisms at the G-protein-coupling site can change the balance of G-protein-coupling profiles to the same drug. For these experiments, we used caerulein, a previously FDA-approved drug (agonist, which closely mimics the endogenous peptide agonist), that was retracted from the market. Nevertheless, it highlights that the susceptibility to adverse reactions to drugs can differ depending on the variant, and that this should be considered in the drug development process (especially, agonists) against receptors that can couple to multiple G-protein families.

The effect of a polymorphism on drug response is more complex than what can be measured in overexpression, cell-based studies, as one needs to consider the entire human physiology. Although drugs (especially agonists) require only certain abundance of the receptor to bring about their maximal response, the expression of a variant allele can (1) result in gain-of-function effects to certain drugs, or (2) alter effector selectivity that will be relevant even in a heterozygous condition with a wild-type allele. In this context, analysis of allele-specific expression data in humans revealed that a significant fraction of the GPCR drug targets are expressed in a mono allelic manner (Figure S5E; p < 1 × 10^−5^). This suggests that the overall abundance of the wild-type (WT) and variant receptor may vary in different heterozygous individuals harboring the same receptor variant. Thus, the presence of a wild-type allele may not always buffer the effects of a variant allele.

**Figure S6:**
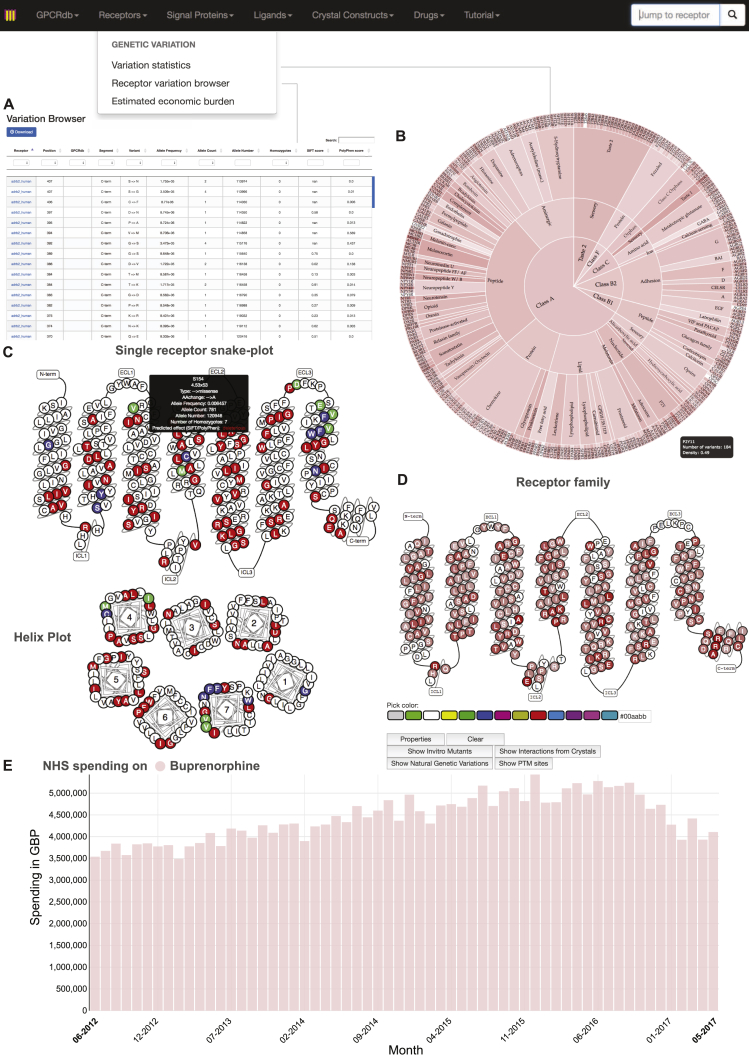
Resource and Tools for the Analysis of Variation Data of GPCR Drug Targets within GPCRdb, Related to Figure 6 Datasets for natural genetic variations comprising of 60,706 individuals have been integrated into the GPCRdb. (A) Sortable variant table is provided for every receptor with more detailed information on the type and nature of each amino acid substitution, information about allele counts and frequency, predicted functional impact scores (SIFT and PolyPhen) and functional site annotation. (B) Genetic variation density (red intensity levels) on a GPCR classification tree (item ‘statistics’). (C) Data points can be visualized for every selected non-olfactory GPCR (missense variants in red shown for adrb2_human) on a snake-like diagram (*top*) or a helix plot (*bottom*) with additional information shown on mouse-over (allele count, allele frequency, amino acid change, number of homozygotes, predicted effects by SIFT and PolyPhen). (D) Missense variants can also be visualized on a consensus snake-like diagram for single families or ligand-types of GPCRs (gradient red for all Class A peptide angiotensin receptors). Each position then gives the number of observed mutations and the list of observed amino acid changes. (E) National Health Service spending data from 2011 to 2017 for 279 FDA-approved drugs. This is shown for Buprenorphine. The natural variation dataset can be downloaded per receptor (www.gpcrdb.org/mutational_landscape/) or accessed programmatically via an extensive API.

Taken together, these observations provide a map of potential pharmacological implications of natural variation in human GPCRs. To facilitate studies on yet-to-be characterized variants, we provide a comprehensive receptor tool and visualization in a new section of the GPCR database (Figure S6; http://www.gpcrdb.org/, “Genetic Variation” tab). This online, interactive platform allows pharmacologists, molecular modelers, clinicians, and anyone interested to select and study the impact of natural variation for any drug target (including those that are/were in clinical trials). This resource is periodically updated to help realize the goals of personalized medicine.

### Drugs and Targets that Are Affected due to Functional Site Variability

**Figure 6:**
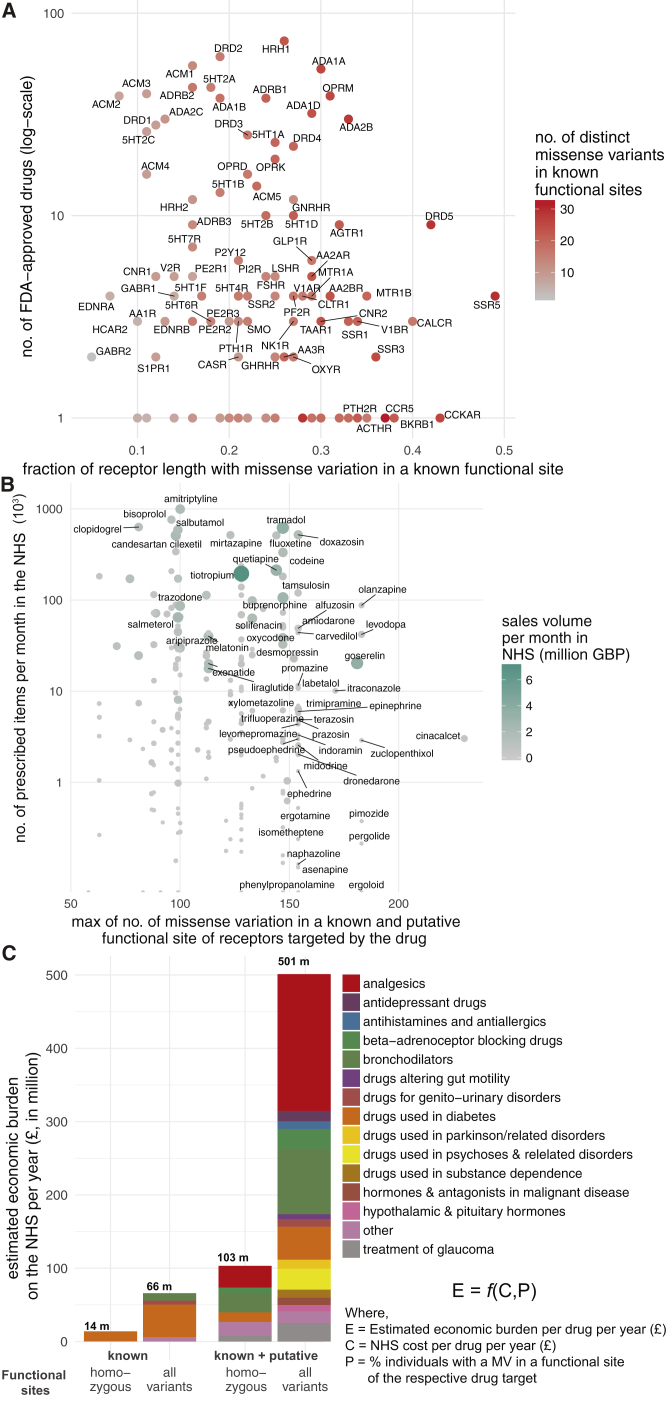
Drugs, GPCR Functional Site Variability, and Associated Economic Impact (A) Number of FDA-approved drugs (y axis, log-scale) against their missense mutation density within known functional sites (x axis) for GPCR drug targets. Color represents the total number of missense variants for each receptor within known functional sites as seen in the ExAC dataset. (B) Number of prescribed items by the National Health Service each month (y axis, log-scale) against the maximum number of missense variations in known and putative functional sites of its therapeutic GPCR target for each FDA-approved drug. UK sales volume is shown in million GBP per month. (C) Estimated economic burden on the National Health Service per year due to ineffective drug prescription (STAR Methods). Please see Table S7 for each drug. See also Figures S6 and S7.

Which receptors have polymorphisms in a significant fraction of their functional sites? For this, we analyzed the ExAC data and identified receptors that show most and least variation in known functional sites. On average, each receptor harbors at least one polymorphism in 23% of the known functional sites (Table S5). Among the highly variable GPCR drug targets are the somatostatin SST_5_ receptor (SSTR5), cholecystokinin A receptor (CCKAR), dopamine D_5_ receptor (DRD5), and the calcitonin receptor (CALCR), all of which display amino acid alterations in more than 40% of the known functional sites (Figure 6A). Thus, for some receptors, the high incidence of variation (i.e., fraction of known functional sites with a MV) makes drug effects more likely, whereas the drug targets with less variability could be expected to maintain the expected drug response. We then developed a score for each drug to rank them based on how likely they are to manifest altered response due to variability in the fraction of known functional site in its drug target(s) (Table S6). We find that ergoloid (for treating cardiovascular disease), olanzapine, and asenapine (both for treating schizophrenia) are drugs whose targets are most variable and thus are more likely to manifest variable response (Table S6).

What fraction of the human population is likely to carry a variant GPCR drug target with a mutation in a known or putative functional site? We find that on average, 3.1% of the 2,504 individuals in the 1000 Genomes Project carry at least one allele with a missense variation in a known functional site in any given GPCR drug target (11.9% in known or putative functional site; Table S5). For instance, over 86% and 69% of the individuals carry at least one allele with a missense mutation in a known functional site in the cannabinoid receptor 2 (CNR2) and glucagon like peptide 1 (GLP1) receptor, respectively, that are targeted by the common antiemetic nabilone, and several anti-diabetic drugs such as exenatide. In line with what we observed for μ-opioid receptor variants, this suggests that a substantial fraction of the population might carry variant receptors and remain healthy but have the potential to display differences in drug response when treated with a drug. Using the genotype information of mutations in known and putative functional sites, we ranked drugs whose targets are most variable (i.e., fraction of population) in their functional sites and thus are more likely to manifest different response (Table S6).

The information and the framework that we describe here can be used to prioritize drugs (Table S6) for pharmacovigilance investigations by regulatory bodies, post-market follow-up studies such as in drug repurposing efforts, as well as in personalizing prescriptions (e.g., dose, treatment regime, etc.). The information on the spectrum and prevalence of receptor variation (Table S5) can also be used for patient stratification of those entering clinical trials in order to maximize success in clinical outcomes.

### Possible Economic Impact of Drug Target Variability

**Figure S7:**
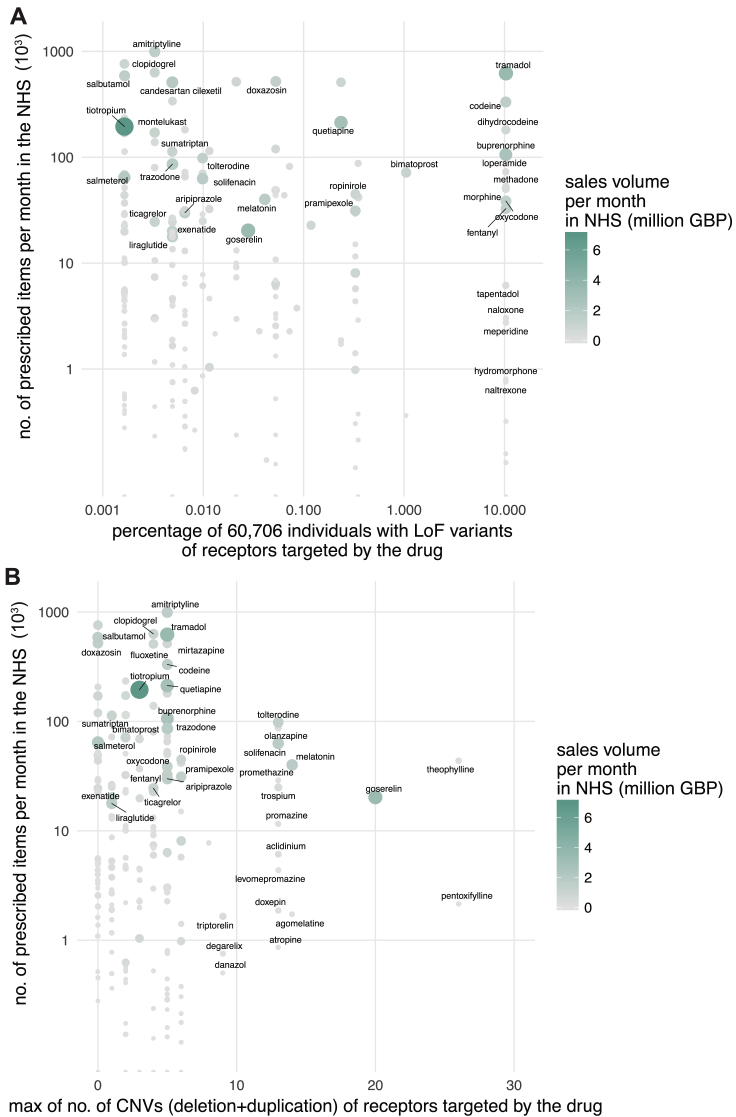
Possible Economic Burden Due to Loss-of-Function and Copy-Number Variation Observed for GPCR Drug Targets, Related to Figure 6 (A–C) For each FDA-approved drug, a pot of the number of prescribed items by the National Health Service each month (*y axis, logarithmic scale in thousands*) against (B) the percentage of individuals with loss of function mutations and (C) maximum number of copy number variations in its therapeutic GPCR target is shown. Sales volume is shown in million GBP per month.

Just in the UK alone, the National Health Service spent ∼1.7 billion pounds in primary care for GPCR targeting drugs in 2016 (∼21% of all drug cost). What is the economic burden of drug cost associated with genetic variation in GPCR drug targets? For this, we investigated all the 279 FDA-approved drugs that are prescribed actively in the UK, the number of prescribed items for a 6-year duration as available in the National Health Service health records (2011–17), and the cost associated with prescriptions. Drugs that are highly prescribed bind to receptors that are polymorphic within known and putative functional sites in the human population (Figure 6B). We find the same trend when considering LoF and CNVs (Figures S7A and S7B). For example, the highly polymorphic drug target, μ-opioid receptor, mediates the effect of morphine, tramadol, codeine, buprenorphine, and fentanyl among other drugs (n = 22) that are prescribed over 4 million times each month and account for sales of more than 432 million British Pounds (GBP) per year just in the UK. Even if a small fraction (7%, from Table S5) of these prescriptions are ineffective or lead to unexpected adverse reactions, such a difference may contribute to a differentially effective treatment outcome and a significant economic burden (an estimate of ∼30 million GBP per year in the UK alone).

To analyze each drug, their GPCR targets, polymorphisms within drug targets, National Health Service prescription and sales data, prescription statistics, affected individuals considering known and putative functional sites as well as homozygosity/heterozygosity, and economic burden estimates, we present an interactive resource at http://gpcrdb.org/mutational_landscape/economicburden. In this simplistic estimate of economic burden (STAR Methods), each prescription is treated as being made for a separate individual due to patient anonymity (i.e., we do not account for the recurrent prescription of the same individual). Furthermore, information about the dose per prescription, and how this has been altered based on patient response is not considered. While we do not independently weigh the variables in our equation, at the level of an individual, the estimates can be higher or lower depending on the exact response due to the polymorphism (i.e., known and putative functional site, homozygosity, heterozygosity, neutral, loss- or gain-of-function effects) and the patient’s alternative management by the National Health Service.

Providing a broad estimate, in the UK, the possible economic burden on the National Health Service due to ineffective prescription of drugs targeting GPCRs could range between 14 million (considering % of individuals with both alleles having a mutation only in known functional sites) and 501 million (considering % of individuals with at least one allele having a mutation in known or putative functional sites) GBP annually (Figure 6C; Table S7; STAR Methods). These estimates do not take into account other sources of economic burden such as hospital prescriptions, nature of the illness (e.g., chronic v/s short-term treatment for acute disease), age of patient (i.e., life-years of treatment), mutations outside of the coding region that affect drug target expression level, associated additional patient care and additional hospital costs in the case of adverse drug reaction. The calculations suggest that polymorphisms in GPCR drug targets may constitute a substantial, unaccounted healthcare expense. Thus, investing efforts on understanding and actively incorporating the effect of drug target polymorphisms and drug response in trials and the clinic has the potential to curtail the recurrent, unaccounted expenditure on public health.

Future studies should carefully collect more information and consider various factors such as the exact effect of heterozygous nature of a receptor polymorphism (i.e., loss of function, gain of function, buffering), the dose and ways in which drugs are prescribed (e.g., repeat prescriptions), efficacy is measured in clinical practice, and drug kinetics (that will vary in different individuals). These factors will be different depending on the drug, receptor, individual, disease, country and medical practice, and need to be considered for each receptor and drug independently to obtain robust estimates of economic burden due to variable drug response. We hope that the resource and analysis platform that we present here and our estimates using a simplistic model will inspire new and novel lines of investigations addressing this essential socio-economic and health problem.

## Discussion

**Figure 7:**
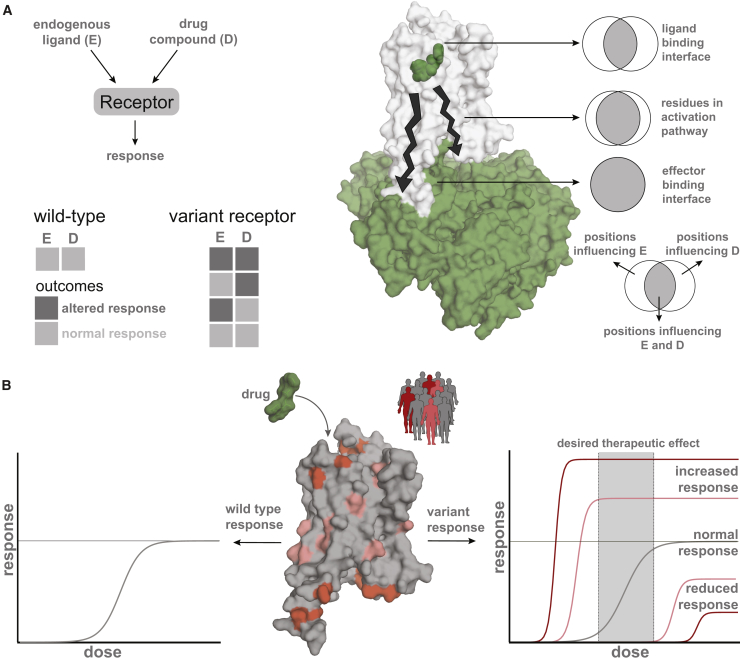
GPCR Variants Can Contribute to Altered Drug Response (A) Receptor response outcome upon binding to an endogenous ligand binding (E) and upon drug treatment (D). A receptor variant may induce phenotype-altering (e.g., disease) perturbations (endogenous ligand) and/or altered drug response (and combinations thereof). The position of the missense variation (top right) renders its effect. Some mutations may have no effect on the binding of the endogenous ligand or the drug and are entirely neutral. Mutations in/near the ligand-binding interface might affect endogenous ligand signaling, drug response, or both. Mutations in the effector-binding interface (G protein/arrestin) most likely affect both endogenous ligand signaling and drug response. (B) Differences in drug response due to different mutations between individuals in a population. The drug target variation spectrum may differently affect individual drug responses by potentially altering ligand potencies and efficacies, receptor conformation, surface expression, and/or biasing signaling. Personalized target sequencing could facilitate prognosis of a patient’s drug response. Additionally, pharmacological characterization of genetic variants that have been cataloged in humans could foster precision prescription. See also Figure S6.

Despite the importance of GPCRs as a major family of drug targets, no receptor variants are included in the labeling information of drugs (FDA, 2014, Thompson et al., 2014, Raimondi et al., 2017). Although most variants have minor consequences and are not described in the literature, even severe adverse effects are estimated to only be reported in 1%–10% of cases (Giacomini et al., 2007), possibly due to a lack of a single post-marketing surveillance system for registering such effects. Additionally, the effects that drugs produce are often confounded by drug-drug interactions due to their combinatorial use, overlap with disease symptoms, and general health status of the patient. Disentangling the contribution of the variation with respect to the disease condition or physiological differences and drug treatment outcomes can help understand how and why certain mutations are buffered under normal physiological conditions in the healthy human population but could cause different responses to drugs (Figure 7A).

While significant efforts are under way for generating large-scale information on genome variation, there is still a huge gap between generating such data and understanding the impact of genome variation (Gallion et al., 2017). Moreover, there are limited experimental resources and efforts for mechanistic understanding of the effects of natural variation. As a first step, detailed characterization of the various polymorphisms in a given GPCR with different doses of FDA-approved drugs from the relevant cells and in native condition can provide insights into predicting individual responses, leading to more precise prescriptions (Figure 7B).

Genetic factors and polymorphisms outside of the coding region can aggravate or alleviate drug response. For instance, synonymous substitutions in exons and mutations within introns can affect splicing patterns; mutations in the inter-genic region can affect regulatory elements and influence the expression levels of drug targets (Ward and Kellis, 2012), both of which can affect drug response. Variation in downstream effectors (e.g., G proteins, arrestin, GRKs) or enzymes that metabolizes drugs (e.g., cytochrome P450) or those that regulate drug uptake can contribute to altered drug reaction (Evans and McLeod, 2003). Other factors such as difference in drug absorption, food effects, tissue distribution, clearance, drug administration compliance, as well as heterogeneity in the disease etiology can all contribute to variability in drug response (Sim and Ingelman-Sundberg, 2011, Lauschke et al., 2017). On the other end of the spectrum, buffering mutations, epistatic interactions, allele-specific expression, and the heterozygous nature of the mutations within an individual might minimize the potential effects of a polymorphism in a drug target under normal drug-free conditions (Lappalainen et al., 2011, Kukurba et al., 2014, Miosge et al., 2015). Thus, it is important to characterize all relevant mutations in the human population, in the right experimental setting, to delineate the direct effects of polymorphisms on drug responses while addressing these issues on a receptor-by-receptor and drug-by-drug basis.

Until recently, drug prescriptions had not been guided by pharmacogenomics, due to the cost and complexity needed to identify, analyze, and interpret genetic variation data (Relling and Evans, 2015). With the advent of sequencing technology and increased international efforts such as the 1000 Genomes Project, ExAC/gnomAD, Psychiatric Genomics Consortium, among others, we are in a unique position with unprecedented access to the vast information on polymorphisms in the healthy and diseased individuals. Characterizing the effects of such variants can be an important step in the design and interpretation of clinical trial studies and could be translated into pre-clinical testing to minimize adverse effects based on pharmacogenomics considerations at a much earlier stage. This can supersede the one-size-fits-all approach in drug treatment, help to prioritize drugs for post-marketing follow-up studies and thus can serve as an important step to personalized optimization of the dosing for already-approved drugs.

In light of the guidelines provided by the FDA for labeling drugs (US Food and Drug Administration, 2013), the findings presented here underscore the importance of characterizing the drug target variants for personalized medicine. Furthermore, access to complete genotype data will be critical to assess the individual risk, prevalence of variation, and their potential impact on adverse drug response. Characterizing variants has the potential to also shed light into receptor biology and help discover principles of how different ligands (e.g., agonists, antagonists, etc.) mediate signaling by binding at allosteric and orthosteric sites. Efforts such as the Clinical Pharmacogenetics Implementation Consortium, Ubiquitous Pharmacogenomics, Pharmacogenomics Research Network, among others are already under way. Dedicated, large-scale efforts, similar to the ENCODE project, for pharmacological characterization of the variants of drug targets would be vital to accomplish this goal. The conceptual framework presented here can be adapted to study other drugs and drug targets such as ion channels, kinases, and nuclear receptors. We hope that the findings from our study and the resource that we present will fuel and equip further advances in personalized medicine.

## STAR★Methods

### Key Resources Table

**Table:** 

REAGENT or RESOURCE	SOURCE	IDENTIFIER
**Chemicals, Peptides, and Recombinant Proteins**		

Endomorphin-1	Tocris	Cat# 1055
Dynorphin A	Abcam	Cat# ab120412
Morphine	Sigma	Cat# M8777
Buprenorphine	Sigma	Cat# B9275
Naloxone	Tocris	Cat# 0599
Caerulein	Toronto Research Chemicals	Cat# C070000
Nano-Glo Luciferase Assay Substrate (furimazine)	Promega	Cat# N1120

**Experimental Models: Cell Lines**		

Human: HEK293T/17 cell	ATCC	Cat# CRL-11268

**Recombinant DNA**		

μ-opioid receptor wt	This paper	N/A
μ-opioid receptor M153V	This paper	N/A
μ-opioid receptor K235M	This paper	N/A
μ-opioid receptor K235N	This paper	N/A
μ-opioid receptor V238I	This paper	N/A
μ-opioid receptor V302I	This paper	N/A
CCKAR wt	This paper	N/A
CCKAR R139I	This paper	N/A
CCKAR Q148E	This paper	N/A
CCKAR R150Q	This paper	N/A
CCKAR R150W	This paper	N/A
CCKAR E234K	This paper	N/A
CCKAR N304H	This paper	N/A
CCKAR V311E	This paper	N/A
Gαo	Hiroshi Itoh	N/A
Gαq	Hiroshi Itoh	N/A
Gαs	Hiroshi Itoh	N/A
Gα13	cDNA Resource Center	Cat# GNA1300001
Venus 156-239 Gβ1	PMID: 19258039	N/A
Venus 1-155 Gγ2	PMID: 19258039	N/A
masGRK3ct-Nluc	PMID: 26628681	N/A
PTX-S1	PMID: 21111235	N/A

**Software and Algorithms**		

GPCRdb	Pándy-Szekeres et al., 2017	https://github.com/protwis/protwis
Python v2.7.13 and v3.4.3	Python Software Foundation	https://www.python.org/; RRID: SCR_008394
R v3.4.1	R Core Team	https://www.r-project.org/
Pandas v0.20.3	Wes McKinney	http://pandas.pydata.org/
Engineered *in vitro* mutations	Munk et al., 2016b	http://www.gpcrdb.org/; RRID: SCR_007419
PyMol v1.8.0.6	Schrodinger	http://pymol.org/2/; RRID: SCR_000305
Arpeggio	Jubb et al., 2017	http://biosig.unimelb.edu.au/arpeggioweb/
SigmaPlot v12.5	Systat Software	http://www.sigmaplot.co.uk/
Prism v6	GraphPad	https://www.graphpad.com/scientific-software/prism/
ChemDraw Professional v16.0	PerkinElmer	http://www.cambridgesoft.com/
Clampfit v10.3	Molecular Devices	http://mdc.custhelp.com/

**Other**		

GPCRdb resource website for the GPCR Pharmacogenomics publication	This paper	https://github.com/AlexanderHauser/protwis/tree/SNP
Custom scripts for analysis	This paper	https://github.com/AlexanderHauser/GPCR-Pharmacogenomics

**Deposited Data**		

Mendeley dataset	This paper	https://doi.org/10.17632/pr5v9t8z36.1

### Contact for Reagent and Resource Sharing

Further information and requests for resources and reagents should be directed to and will be fulfilled by the Lead Contact, M. Madan Babu (madanm@mrc-lmb.cam.ac.uk).

### Experimental Model and Subject Details

#### Cell Lines and Tissue Culture

HEK293T/17 cells were grown in culture medium (Dulbecco’s modified Eagle’s medium supplemented with 10% fetal bovine serum, MEM non-essential amino acids (Life Technologies), 1 mM sodium pyruvate, and antibiotics (100 units/mL penicillin and 100 μg/mL streptomycin) at 37°C in a humidified incubator containing 5% CO_2_. This cell line is derived from a female and was purchased from ATCC.

### Method Details

#### Use of generic residue numbering systems to compare GPCR positions

In order to make the findings presented here applicable to all GPCRs, the GPCRdb numbering system (http://gpcrdb.org) was used throughout this study (Isberg et al., 2015). The structure-based GPCRdb numbering scheme is an adaption of the sequence-based Ballesteros-Weinstein scheme with corrections for helix bulges and constrictions. GPCRdb numbers are distinguished by a unique separator x and may be used alone, e.g., 5x47, or together with one of the sequence-based schemes (such as Ballesteros-Weinstein), e.g., 5.46x47. Cross-class comparisons of generic residue positions (note, that e.g., 6x50 in Class A does not correspond to 6x50 in Class B) were conducted according to a class residue translation table (Isberg et al., 2015). The offset to the Class A x.50 to the corresponding Class B position can be deduced from following offsets: TM1-7: 4, 7, 4, 0, −4, 5, 4. Class A to C is translated with the following offset for TM1-7: 4, −4, 4, −10, 0, 2, −5. Class A to F is translated by TM1-7: −3, −1, 0, 0, 4, −1, 0. For example, the Class C 4x50 position corresponds to the 4x60 position in Class A receptors.

#### Datasets

##### FDA-approved drugs and their GPCR targets

All FDA-approved drugs and agents that are/were in clinical trials that target GPCRs were obtained from GPCRdb (Hauser et al., 2017). In this list, every single FDA-approved drug-receptor interaction has a source reference article obtained via DrugBank (ver. 5.0.7) (Law et al., 2014) and literature search. The respective references (PubMed IDs) are available as annotation. Other annotations include primary and secondary targets of approved drugs and additional targets that reached clinical trials. For the latter, information about whether it is still in clinical trials (“ongoing”) or terminated is provided. Approved agents that are close analogs of the equivalent endogenous ligands are noted. The list of drug-GPCR interactions is provided in Table S1. Please see www.gpcrdb.org/drugs/drugbrowser (for all drug-receptor pairings) and www.gpcrdb.org/drugs/drugmapping (for a GPCR drug target overview). The dataset from Hauser et al. (Hauser et al., 2017) contains 475 FDA-approved drugs that target 108 GPCRs. Additionally, it contains 532 agents in clinical trials (discontinued and ongoing) targeting a total of 165 receptors. Of these, 66 receptors do not have any FDA-approved drug (i.e., not part of the 108 GPCR drug targets).

##### Genotype data

To estimate the number of coding loci with missense mutations (with respect to the reference human genome GRCh37/hg19) within the GPCR drug targets in an individual, we obtained genotype data for 2,504 individuals from the 1000 Genomes Project (Auton et al., 2015). Each of the genomes of the 2,504 individuals was separately investigated for missense variants in GPCR drug targets and their clinical association with altered drug response in literature.

##### De novo mutations data

To estimate the *de novo* missense mutation rate within GPCR drug targets, we obtained *de novo* mutations from 1,762 control trios (without any reported pathological conditions) compiled from ten different studies from denovo-db (Turner et al., 2017).

##### Genetic variation data

We retrieved natural genetic variation data for all GPCRs from the Exome Aggregation Consortium (ExAC), which compiled exome-sequencing data from various large-scale cohorts spanning 60,706 unrelated individuals from 6 distinct human populations. We extracted polymorphism data for the coding-region of the 108 GPCR drug targets and GPCRs that reached clinical trials (n = 66) via their respective Ensembl transcript IDs as used in the GPCRdb **(**Table S5). Minor Allele frequencies (MAFs) were calculated as the ratio of allele counts of the less frequent allele to the total number of alleles at that locus. Each variant was mapped to its respective structural segment (e.g., transmembrane regions, intra-cellular loops, extra-cellular loops, etc.), generic residue position (i.e., GPCRdb numbering), receptor class, ligand-type and family classification according to the GuideToPharmacology (Southan et al., 2016) and GPCRdb (Munk et al., 2016a) using custom R and python pandas scripts.

Densities (number of MVs normalized to the protein length) were calculated using the respective protein length. Missense variants were categorised into “changed” and “similar” depending on whether the amino acid substitution switched defined categories such as hydrophobic (‘A’, ‘C’, ‘F’, ‘I’, ‘L’, ‘M’, ‘V’, ‘W’, ‘Y’), aromatic (‘F’, ‘H’, ‘W’, ‘Y’), polar uncharged (‘S’, ‘T’,’N’, ‘Q’), helix breakers (‘P’, ‘G’), negative (‘D’, ‘E’) and positive (‘H’, ‘K’, ‘R’) or remained within the same category. Loss of function (LoF) mutations was extracted by filtering for essential splice sites, gained stop codon or frameshift mutations. As no genotype data were available, to calculate the number of individuals with a LoF mutation, we estimated the minimum number of individuals with LoF mutations. For this, we extracted positions within a GPCR drug target with the highest allele count for LoF mutations and corrected for homozygous individuals (i.e., the number of homozygotes were subtracted from the total allele count to arrive at the number of heterozygous individuals). This number (minimum population frequency) reflects the minimum number of heterozygous individuals, who carry at least one LoF mutation in a GPCR drug target. Copy number variations (CNVs) were analyzed from 59,898 human exomes for all GPCR drug targets (Ruderfer et al., 2016). For a total of 82 receptor genes out of the 108 GPCR drug targets, CNVs could be confidently called. We calculated the z-score for each category (number of missense variants, missense variant density, number of loss of functions, loss of function minimum population frequency, number of deletions and number of duplications) by

$$z-score=\frac{x-\mu }{\sigma }$$

where:

x is the observed value for the category considered.
μ is the mean of the 108 GPCR drug targets in a given category.
σ is the standard deviation of the 108 GPCR drug targets in a given category.

The absolute value of the z-score represents the distance between the individual observed value and the population mean in units of standard deviation. We then used hierarchical clustering ‘heatmap.2′ from ‘gplots’ in R to identify receptors that are highly polymorphic in multiple mutation categories (Figure S1B).

#### Estimation of putative functional impact of variants in GPCR drug targets

An analysis of the putative functional impact of variants was obtained using SIFT (Ng and Henikoff, 2003) (sorting intolerant from tolerant) and PolyPhen (Adzhubei et al., 2013) (polymorphism phenotyping). A missense variant position was classified as a putative functional site if SIFT (0-1; 0-0.05: deleterious, > 0.05: tolerated) or PolyPhen outcome (0-1; 0-0.1: benign, 0.1-0.2: possibly damaging, > 0.2: probably damaging) predicted a deleterious or possibly damaging outcome. This resulted in the identification of 9,522 variants (referred to as putative functional sites).

#### Engineered in vitro mutations

Data on 28,779 experimentally characterized *in vitro* mutations was retrieved from the GPCRdb (Pándy-Szekeres et al., 2017). From this dataset, we extracted 9240 mutants for human receptor constructs and with reported effect values on ligand affinity/potency. This includes experimental values for different ligands for a total of 1,996 distinct receptor positions. Additionally, all experimental data obtained by investigating orthologous receptors from related species such as mouse, rat, hamster, dog, pig and rhesus macaque were included if the corresponding generic structural-alignment position in the human receptor was identical in amino acid (Isberg et al., 2015). The combined dataset comprised of 13,223 mutants in 68 distinct receptors and a total of 928 unique ligands. We then looked for identical amino acid substitutions (same receptor, position, wild-type and mutant amino acid) that mimicked a naturally occurring variant in the ExAC dataset for the 108 GPCR drug targets. In cases where there were multiple data points, we considered maximum fold changes. (Table S4). A cut-off of 5-fold for the maximal absolute fold changes was applied for experimental *in vitro* support of impact on drug response (Munk et al., 2016b).

#### Clinical annotations

Clinical information about missense variant-drug pairs with reported changes in efficacy, dosage or toxicity/ADR were manually curated from the literature and PharmGKB, which aggregates the impact of human genetic variation on drug response (https://www.pharmgkb.org/). We obtained the level of evidence for each association as listed in PharmGKB. If it was not available, we assigned the evidence level based on PharmGKB criteria (https://www.pharmgkb.org/page/clinAnnLevels), so as to guide the reader in terms of the evidence that is available for a particular clinical association with a receptor variant. All figures and text referring to clinical associations have been limited to a level of evidence of 3 or above (3, 2 and 1). In this annotation scheme, 4 is the lowest level of evidence and 1 is the highest level of evidence. Disease annotations were extracted from the indications in the drugs@FDA database (https://www.accessdata.fda.gov/scripts/cder/daf/**)** or the actual study that reported the altered drug response. Higher disease ontology categories were assigned using the Experimental Factor Ontology (EFO) retrieved from the Open Targets database (Koscielny et al., 2017). Corresponding positions and amino acid substitutions of reported SNP identifiers were retrieved using BioMart for the “canonical transcripts” as stored in GPCRdb (Table S5) (Smedley et al., 2015). Network analysis between disease categories, drugs and missense variants were done using Cytoscape 3.4.

#### Allele-specific expression data

We obtained information on allele-specific expression of genes from published literature (Savova et al., 2016). Briefly, genes were classified as those with monoallelic and biallelic expression, by integrating gene-expression data and specific chromatin signatures of gene expression (co-occurrence of silencing mark on Histone H3K27me3 and active mark H3K36me3 on the gene body) in six different cell types. We assessed for enrichment of GPCR drug targets among genes with monoallelic expression using permutation test.

#### Fraction of receptor length with a polymorphism or population with variant receptor

For each of the GPCR drug targets we calculated: (i) the ratio of receptor length with missense variation in a known functional sites per GPCR drug target using the ExAC data (Table S5) and (ii) the fraction of affected individuals in the human population (n = 2,504; based on the 1000 Genomes Project dataset, Table S5). The fraction of affected individuals was calculated using four different criteria by considering individuals who have a variation in (i) known functional sites in both alleles (homozygous), (ii) known functional sites in at least one allele (i.e., homozygous and heterozygous), (iii) known or putative functional sites in both alleles (homozygous), and (iv) known or putative functional sites in at least one allele (i.e., homozygous and heterozygous). Known functional sites include ligand binding, effector binding, post-translational modification site, sodium binding site and micro-switches. Putative functional sites include those predicted to be deleterious based on SIFT or PolyPhen.

#### Drug scores for prioritization

We developed a score for each FDA-approved drug (n = 475) to rank them based on how likely they are to manifest altered response due to the prevalence of known functional site variability of its target(s) in the human population.

The drug score (Table S6) based on the fraction of known functional site that are polymorphic in a drug target using the ExAC data was calculated by:

$$variability\;score\;for\;a\;drug,S_{polymorphic}=\sum fraction\;of\;known\;functional\;sites\;that\;are\;polymorphic\;for\;each\;receptor\;targeted\;by\;the\;drug$$

The drug score (Table S6**)** based on prevalence of affected individual (i.e., 1000 Genomes Project) was calculated by:

$$variability\;score\;for\;a\;drug,S_{affected\%}=fraction\;of\;affected\;individuals\;with\;a\;MV\;in\;a\;functional\;site\;of\;the\;respective\;drug\;target\left(s\right)$$

The fraction of affected individuals was calculated using four different criteria by considering individuals who have a variation in (i) known functional sites in both alleles (homozygous), which is the most conservative, (ii) known functional sites in at least one allele (i.e., homozygous and heterozygous), (iii) known or putative functional sites in both alleles (homozygous), and (iv) known or putative functional sites in at least one allele (i.e., homozygous and heterozygous), which is the least conservative.

#### National Health Service prescription data

Every month, the National Health Service (NHS) in the UK publishes anonymised data about the drugs prescribed by general practitioners. National Health Service data were retrieved from openprescribing.net (DataLab-EBM, 2017) (08/2017) for the list of drugs targeting GPCRs and mapped back to their reported target of therapeutic action. From the 475 queried FDA-approved drugs, data were available for 279 drugs targeting 92 distinct GPCRs (not all FDA-approved drugs are prescribed in the UK due to alternative treatments). Items are the number of times the drug appeared on a prescription form that month (defined by National Health Service Digital as “A prescription item is a single supply of a medicine, dressing or appliance written on a prescription form”). The actual cost is the estimated cost to the National Health Service, which is usually lower than Net Ingredient Cost (“the basic price of a drug, i.e. the price listed in the Drug Tariff or price lists”). Openprescribing.net provides the actual cost by subtracting the average percentage discount per item received by pharmacists based on the previous month from the Net Ingredient Cost, but adding in the value of a container allowance for each prescription item (DataLab-EBM, 2017). Total National Health Service spending was aggregated over each month of 2016 and compared with the total GPCR-targeting drugs to calculate the share for the GPCR targeting drugs (Figure 1). Indications were grouped according to the British National Formulary (BNF), which is a reference book containing the standard list of medicines prescribed in the UK and also includes information on indications, dosage and side effects.

#### Estimation of economic burden

The economic burden estimate was calculated using the following formula:

estimatedeconomicburdenperdrug(£)=averageNHScostperdrugperyear(£)x

%individualswithaMVinafunctionalsiteoftherespectivedrugtargets

where:

• The average National Health Service cost is the average yearly cost over a 4-year period (2013-2016) per GPCR targeting drug that is listed (n = 279). 2012 and 2017 have partial sales data and were not considered.
• % Individuals is the percentage of affected individuals with a missense variant in a functional site of the respective drug target(s) (n = 2,504 individuals from 1000 Genomes Project genotype data as a representative for the UK population; this data includes non-Caucasian populations as well) (Table S5).
• The % of affected individuals was calculated using four different criteria by considering individuals who have a variation in (i) known functional sites in both alleles (homozygous), which is the most conservative, (ii) known functional sites in at least one allele (i.e., homozygous and heterozygous), (iii) known or putative functional sites in both alleles (homozygous), and (iv) known or putative functional sites in at least one allele (i.e., homozygous and heterozygous), which is the least conservative.
• Known functional sites include ligand binding, effector binding, post-translational modification site, sodium binding site and micro-switches. Putative functional site include those predicted to be deleterious based on SIFT or PolyPhen (see above).

More specifically, for each drug we collected the respective targets and computed economic burden using the following four criteria above: considering (i) % individuals with homozygous alleles in known functional sites, (ii) % individuals with at least one variant allele in a known functional site, (iii) % individuals with homozygous alleles in known or putative functional sites and (iv) % individuals with at least one variant allele in a known or putative functional sites.

For these estimates, we have incorporated the following considerations (below). The economic burden estimates will vary if one scales/factors these variables differently:

1. We have considered that each prescription (National Health Service data) is made for a unique individual, due to patient anonymity. Furthermore, information about the dose per prescription, and how this has been altered based on patient response is not explicitly modeled.
2. The effect of known and putative site polymorphisms as well as homozygous/heterozygous conditions are all treated the same way. One could also obtain estimates by weighing these variables differently on a case-by-case basis for each receptor/drug.
3. The focus has been prescription only from GPs. There might be significant additions to the economic burden if one also considers hospital prescriptions.
4. We used the data from 1000 Genomes Project as representative of the UK population, which may vary depending on the receptor.
5. We have not explicitly modeled the age, gender, nature of illness (chronic v/s short-term) and mutations in non-coding regions, which may affect expression level of drug targets.

#### Interaction interface, functional, and structural site assignment

##### Post-translational modification sites

We obtained publicly available experimental data on post-translational modification sites for the GPCR drug targets (including Ubiquitylation, Phosphorylation, Palmitoylation, Hydroxylation, N/O-linked Glycosylation, Sulfation, Prenylation, ADP-ribosylation, Methylation, Sumoylation, Acetylation, Disulfide bond and S-Nitrosylation) from dbPTM (Huang et al., 2016). Additionally, all post-translational modification sites from PhosphoSitePlus (03/2017) from low-throughput experimental techniques, proteomic mass spectrometry and shotgun proteomic experiments were collected and combined with the dbPTM dataset (Hornbeck et al., 2015) (Table S4). Each post-translational modification site was cross-validated for identical amino acids in both dbPTM and its corresponding GPCRdb wild-type amino acid. For each receptor, we then obtained the post-translational modification sites with reported missense variation.

##### Generic transmembrane ligand-interacting sites

Ligand interaction sites were extracted from all 196 available receptor-ligand crystal structure complexes featuring 43 unique GPCR ligand-receptor complexes from *Chordate* organisms and higher (excluding organisms such as *Herpesvirus 5* and *Todarodes pacificus*). The union of ligand-interacting positions were aggregated for each of the 42 unique crystallized receptors including aromatic, polar, and hydrophobic interactions within 4.5 Å of the co-crystallized ligand (Pándy-Szekeres et al., 2017, Munk et al., 2016b). This led to a total set of 457 ligand-binding residues. Ligand binding site definition for receptors without structural information have been inferred from the receptor family level (based on NC-IUPHAR receptor family nomenclature obtained from GPCRdb) for which structural information was available (e.g., dopamine D_5_ receptor ligand-binding site is inherited from dopamine D_3_ receptor). To infer the ligand binding site from the crystallized receptor, we used only positions that have a generic numbering in the GPCRdb numbering scheme (Isberg et al., 2015), which excludes ligand-interacting positions for most of the loops (three ECL2 positions, including 34x52 at the top of the 7TM ligand cavity, are included). For ortholog receptors (e.g., mouse, rat, bovine), we inferred generic positions only if the corresponding human residue is identical to the ortholog receptor. This allowed ligand-binding pocket characterization for 24 families and a total of 139 receptors. We provide annotation as to whether a position is a known ligand-binding site (LB) from a crystallized structure or an inferred one from a related structure of the same receptor family in Table S4.

##### Arrestin binding interface

All available GPCR-arrestin complexes (PDB: 4ZWJ, PDB: 4PXF, PDB: 5DGY, all are Class A rhodopsin structures) were retrieved from the PDB and visualized using PyMol. The inter GPCR-arrestin residue contact network (RCN) for these structures was computed using Arpeggio (Jubb et al., 2017) with the maximum range of interaction set to 4.5 Å. The union of all interacting residue positions was taken and corresponding residues (with same GPCRdb numbers) were retrieved from the GPCRdb WebServices. This led to the identification of 29 arrestin contacting positions that were used for the effector interaction site analysis. Arrestin residue positions were applied to other classes by their corresponding generic position (see *Use of common residue numbering systems to compare GPCR positions*).

##### G protein binding interface

The inter GPCR-G protein residue contact network was generated as described for arrestin. For the class A interface, the β2AR-Gs (PDB: 3SN6), A_2A_-Gs_mini_ (PDB: 5G53) and Rhodopsin-Gt-C-peptide (PDB: 3DQB, PDB: 3PQR, PDB: 4A4M) were used. This led to the identification of a total of 33 G-protein interacting positions on the receptor. The class B GPCR-G protein interface was calculated using the Calcitonin Receptor-Gs (PDB: 5UZ7) and the GLP-1-Gs (PDB: 5VAI) structures. For class C and class F receptors, the union of both class A and B interface positions were considered as the G protein coupling positions.

##### Microswitches

Residues involved in mediating the conformational transition during activation (microswitches) were obtained from the literature (Trzaskowski et al., 2012). These include: D/E^3x49^, K/Y^7x43^, R^3x50^, F^5x47^, Y^5x58^, E^6x30^, T^6x34^, W^6x48^, P^6x50^, N^7x49^, P^7x50^, Y^7x53^, I^3x40^. Additionally, we considered the “P-I-F” motif (P^5x50^, I^3x40^ and F^6x44^) as an important motif for conformational rearrangements upon receptor activation (Wacker et al., 2013). The positions 3x46, 6x37 and 7x53 were also considered as microswitches due to their importance in structural rearrangements (Venkatakrishnan et al., 2016). Micro-switches were only considered for Class A receptors.

##### Sodium ion pocket

Residue positions (n = 15) of the sodium pocket were extracted from Katritch et al. (Katritch et al., 2014) and comprised of N^1x50^, V^1x53^, L^2x46^, A^2x47^, A^2x49^, D^2x50^, S^3x39^, L^3x43^, F^6x44^, W^6x48^, N^7x45^, S^7x46^, N^7x49^, P^7x50^, Y^7x53^. The Na^+^ pocket was only considered for Class A receptors.

##### Structural site assignments

Segments of structural sites were extracted using class-specific multiple sequence alignments. Helix segments were assigned based on a residue independent generic numbering position as described in the GPCRdb (e.g., 1x50 - > TM1). Residues falling between transmembrane regions were assigned to their respective loop region (extracellular and intracellular loops 1-3). Residues before TM1 and after Helix 8 were assigned C-terminal and N-terminal, respectively.

#### Pharmacological validation

##### Selection criteria for in vitro variants

We performed functional analysis and pharmacological validation of mutations in two GPCRs for the ligand binding (OPRM) and G protein binding (CCKAR). We selected the μ-opioid receptor, due to its importance in analgesia, physical dependence and respiratory depression. Additionally, μ-opioid receptor is targeted by nearly 40 FDA-approved drugs and is one of the highly polymorphic receptor. Specifically, a structural analysis identified 5 variants of potentially damaging or altering effects upon drug binding. These include M153V^3x36^, K235M^5.39x40^, K235N^5.39x40^, V238I^5.42x43^ and V302I^6x55^. For the Cholecystokinin A receptor (CCKAR) we investigated the effect of polymorphisms at the G protein binding interface, which is known to couple to several G proteins to produce its physiological effects (Dufresne et al., 2006). We selected 7 variants at positions, which are potentially important to interact with the G protein, that were predicted to be deleterious in SIFT and PolyPhen and all mutations changed their residue properties (see *Genetic variation data*). These include R139I^3x50^, Q148E^34x52^, R150Q^34x54^, R150W^34x54^, E243K^5x72^, N304H^6x26^, V311E^6x33^. Experimental outcome of all selected variants is provided in Figure S5.

##### Genetic constructs

Human wild-type and mutant receptors were synthesized and cloned into pcDNA3.1(+) by GenScript. pCMV5 plasmids encoding Gαo, Gαq, and Gαs were gifts from Dr. Hiroshi Itoh (Nara Institute of Science and Technology, Japan). Plasmids encoding Venus 156-239-Gγ1, and Venus 1-155-Gγ2 were gifts from Dr. Nevin A. Lambert (Augusta University, USA) (Hollins et al., 2009). masGRK3ct-Nluc construct were generated as reported previously (Masuho et al., 2015a). PTX-S1 in mammalian expression vector was kindly provided by Dr. Eitan Reuveny (Weizmann Institute of Science, Israel) (Raveh et al., 2010).

##### Transient transfection

We coated 6-cm culture dishes during incubation for 10 min at 37°C with 2.5 mL of Matrigel solution (approximately 10 μg/mL growth factor-reduced Matrigel (BD Biosciences) in culture medium). Cells were seeded into the 6-cm dishes containing Matrigel solution at a density of 4 × 10^6^ cells/dish. After 4 hr, expression constructs (total 10 μg/dish) were transfected into the cells using PLUS (10 μl/dish) and Lipofectamine LTX (12 μl/dish) reagents. Venus 156-239-Gγ1 (0.42 μg), Venus 1-155-Gγ2 (0.42 μg), and masGRK3ct-Nluc (0.42 μg) was transfected with different amount of Gα and receptor constructs. μ-opioid receptor (2.52 μg), cholecystoninin A receptor (2.52 μg), GαoA (0.42 μg), Gαq (0.84 μg), Gαs (2.52 μg), or Gα13 (1.68 μg) was used. Empty vector pcDNA3.1(+) was used to normalize the total amount of transfected plasmid DNA. PTX-S1 (0.42 μg) was transfected with Gαq, Gαs, and Gα13 to inhibit possible coupling to endogenous Gα belonging to Gi/o subfamily.

##### BRET assay for monitoring G protein activity

BRET experiments were performed as previously reported with slight modifications (Masuho et al., 2015a, Masuho et al., 2015b). Sixteen to twenty-four hours post-transfection, HEK293T/17 cells were washed once with BRET buffer (PBS containing 0.5 mM MgCl_2_ and 0.1% glucose) and detached by pipetting with BRET buffer gently. Cells were harvested with centrifugation at 500 g for 5 min and resuspended in BRET buffer. Approximately 50,000 to 100,000 cells per well were distributed in 96-well flat-bottomed white microplates (Greiner Bio-One). The Nluc substrate, furimazine, were purchased from Promega and used according to the manufacturer’s instruction. BRET measurements were performed using a microplate reader (POLARstar Omega or PHERAstar *FSX*, BMG Labtech) equipped with two emission photomultiplier tubes. All measurements were performed at room temperature. The BRET signal is determined by calculating the ratio of the light emitted by Venus-Gβ1γ2 (535 nm with a 30-nm band path width) over the light emitted by masGRK3ct-Nluc (475 nm with a 30-nm band path width). The average baseline value recorded before agonist stimulation was subtracted from BRET signal values, and the resulting difference (ΔBRET ratio) was plotted as traces. The transiently transfected cells were stimulated by endomorphin-1 (100 μM), morphine (100 μM), buprenorphine (10 μM), naloxone (100 μM) for μ-opioid receptor or caerulein (30 μM) for CCKAR at 5 s. No agonist-induced activation of G proteins was detected from the negative control that omitted CCKAR. Each trace represents the mean of the responses measured in three or six wells. The maximal value recorded upon agonist stimulation was reported as *R*_Max_. The activation rate constants (*k*_ON_) were obtained by fitting a single exponential curve to the traces with Clampfit ver. 10.3 software (Molecular Devices).

### Quantification and Statistical Analysis

#### Pharmacological validation

Statistical analysis to analyze the experimental data was performed with GraphPad Prism 6. Statistical parameters are reported in Figure Legends. All graphs pertaining to the experimental data were made with SigmaPlot 12.5.

### Estimation of statistical significance

#### Enrichment of receptors with allele-specific expression

Enrichment of GPCRs among genes with monoallelic expression was assessed with permutation tests by performing 100,000 randomizations. In each randomization, each GPCR was replaced with a random gene and the number of such randomly obtained genes that overlapped with genes with monoallelic expression was noted. From the random distribution, we computed the Z-score, which captures the distance of the actual number of observations (e.g., GPCRs with monoallelic expression) to the mean of random expectation in terms of the number of standard deviations. We estimated p value as the ratio of the number of simulations where the random observations were greater than or equal to the number of actually observed values to the total number of randomizations (100,000).

#### Enrichment of MVs in known functional site

To assess for enrichment of MVs affecting known functional sites, we obtained a list of random sites with similar number of sites with MV for all GPCRs and then counted the number of random sites that overlapped with known functional sites. We performed 100,000 such randomizations to obtain a distribution of random expectations. Z-score and p values were estimated as described above.

#### Statistical significance for overlap between known and putative functional sites

For assessing whether the overlap between known and putative functional sites (deciphered from SIFT and PolyPhen predictions) was significantly higher than expected by chance, we randomly replaced each known functional site with a random site with a missense mutation in GPCRs and noted how many of these random mutant sites overlapped with the putative functional sites. We performed 100,000 such randomizations and obtained a distribution of expected overlap by chance. Z-score and p values were estimated as described above.

#### Statistical significance for putative functional sites in different structural segments

We estimated statistical significance of differences in the distribution of putative functional sites and uncharacterized sites in different structural segments of GPCRs using the non-parametric Wilcoxon rank sum test (Figures S4C**;** *absolute counts* and S4D**;** *normalized for segment length*).

### Data and Software Availability

#### Data and code availability

All relevant data are integrated into the web resource in GPCRdb (Pándy-Szekeres et al., 2017) (www.gpcrdb.org) and can also be obtained from GitHub (https://github.com/protwis/gpcrdb_data). All data that support the findings of this study have been provided as Tables S1, S2, S3, S4, S5, S6, and S7 as were deposited in Mendeley Data (https://doi.org/10.17632/pr5v9t8z36.1). The open-source code can be obtained from GitHub (https://github.com/protwis/protwis). In-house written scripts can be obtained from GitHub (https://github.com/AlexanderHauser/GPCR-Pharmacogenomics). For specific data/script requests please contact the lead contact.

### Additional Resources

#### Missense variation mapping of specific receptors

To analyze and characterize the extent of genetic variation, we incorporated all missense variations and functional annotations into the framework of the GPCRdb (http://www.gpcrdb.org/) that allows researchers to map all missense variants for a selected receptor, using NC-IUPHAR receptor nomenclature, onto a snake–like diagram and helix-boxplot of the selected receptor residue topologies. Further, information regarding amino acid substitution, allele counts, allele frequencies, functional annotations, SIFT/PolyPhen scores and heterozygosity for each individual position is provided (www.gpcrdb.org/mutational_landscape/). A statistic page is presented for an overview of genetic variation in GPCR drug targets (www.gpcrdb.org/mutational_landscape/statistics). Possible economic impact of drug target variability can be analyzed and estimated for individual drugs and different groups of drugs via http://www.gpcrdb.org/mutational_landscape/economicburden. The National Health Service spending on each drug can be obtained from the drug-receptor pairing page, which includes specific information of the drug such as indication, target family, target category (primary/secondary), status, drug type, mechanism of action and references (www.gpcrdb.org/drugs/drugbrowser). The natural variation dataset can be downloaded in full, accessed via an extensive API (http://gpcrdb.org/services/reference/), or searched and browsed via a web interface.

## Author Contributions

A.S.H. and M.M.B. designed the project, analyzed the data, interpreted the results, and wrote the manuscript. All authors read and provided inputs on the manuscript. A.S.H. compiled the datasets, wrote scripts, implemented and performed the calculations, made figures, and developed the web services. S.C. was involved in the compilation of variation data, permutation test, and calculation of allele-specific expression and making the relevant figures. L.J.J. curated clinical associations. I.M. and K.A.M. performed the experiments, analyzed the data, and contributed to writing the experimental section of the manuscript. D.E.G. provided critical inputs on the project, manuscript writing, and data analysis. M.M.B. initiated, managed, and set the direction of research for the project.

## Supplemental Information

Table S1. Drug-GPCR Interaction Data, Related to Figures 1, 2, 3, 4, 5, 6, and 7

Table S2. Reported Receptor Variant-Drug Response Associations, Related to Figures 2 and 4

Table S3. GPCR Drug Targets Variant Summary and *Z* Scores, Related to Figures 3, S1, and S2

Table S4. Complete Variant Table of Clinically Relevant GPCR and Functional Annotations, Related to Figures 1, 2, 3, 4, 5, 6, and 7

Table S5. Number of Affected Individuals and Fraction of Mutations in Known Functional Sites in GPCR Drug Targets and ID Mapping, Related to Figures 6 and S4

Table S6. Variability Score of GPCR Drugs and for a Drug in Functional Sites in GPCR Drug Targets, S-Polymorphic, and S-Affected%, Related to Figure 6

Table S7. Estimated Economic Burden, Related to Figures 6 and S6
